# Conditional T and NK cell antagonism by a giant and highly conserved orthopoxvirus virulence factor

**DOI:** 10.21203/rs.3.rs-8672980/v1

**Published:** 2026-02-04

**Authors:** Stephen D. Carro, Emma J. Hedgepeth, Candy Lucero-Sanchez, Mary K. Heard, Angela R. Corrigan, Heejoon M. Shin, Elise M. Peauroi, Laurence C. Eisenlohr

**Affiliations:** 1 Department of Pathology and Laboratory Medicine, Perelman School of Medicine, University of Pennsylvania; Philadelphia, PA 19104, USA; 2 Department of Pathology and Laboratory Medicine, Children’s Hospital of Philadelphia; Philadelphia, PA 19104, USA; 3 Department of Microbiology, Perelman School of Medicine, University of Pennsylvania; Philadelphia, PA 19104, USA; 4 Department of Chemistry, School of Arts and Sciences, University of Pennsylvania; Philadelphia, PA 19104, USA; 5 Lead contact

## Abstract

Orthopoxviruses, including variola and monkeypox, have long ravaged human populations for reasons that remain unclear. Members of the highly conserved B22 protein family are notable for their extreme virulence via targeting of multiple host defenses. C15, the B22 protein of ectromelia (murine model for smallpox), is known to target NK cells and CD4+ T cells, and, as shown here, also CD8+ T cells. Unexpectedly, in C57Bl/6 mice, T cell responses were larger, more functional, and phenotypically enhanced in the face of C15 expression. cDC1-mediated cross-presentation contributed to enhanced CD8+ T cell responses, but the primary contributor was C15-mediated antagonism of NK cell-dependent viral control, and single cell analysis identified a potential signaling scaffold downstream of C15 inhibitory activity. Conversely, in BALB/c mice, which mount suboptimal NK cell responses, T cells were more prominently inhibited. These studies introduce the concept of conditional immunomodulation dictated by the immunocompetence profile of the host.

Orthopoxviruses (OPXVs) are a genus of mammalian-tropic viruses that exhibit a broad host range, induce severe pathology, and are a significant public health burden^1,2^. Although the most prominent OPXV, variola (the causative agent of smallpox), was eradicated in 1980, the global human-to-human spread of mpox (MPXV)^3^ as well as the emergence of novel OPXV species^4–6^ highlight the substantial risk OPXVs continue to pose to the general population. Furthermore, the 2022–23 outbreak of Clade IIB mpox revealed significant limitations of current anti-OPXV therapeutics, including sub-optimal vaccine efficacy^7–10^ and ineffectiveness of current FDA-approved antivirals^11–14^. Generally, OPXV virulence is attributed to the abundant immunomodulatory proteins encoded within their genomes, but many of these proteins are understudied and are also not shared across the entire genus, creating barriers to the establishment of general principles of OPXV virulence.

Notable among OPXV immunomodulatory proteins is the B22 family of proteins^15–19^. Encoded by the largest open reading frames (ORFs) in OPXV genomes, B22 proteins are highly conserved in all OPXVs except for vaccinia^15^, which was used to develop the smallpox and mpox vaccines due to its dampened virulence^20^. Initial *in vitro* studies found that B22 proteins, including those from monkeypox, cowpox (CPXV), and variola, are potent inhibitors of T cell receptor (TCR)-dependent activation^15^. Furthermore, studies in non-human primates demonstrated that infection with MPXV lacking its B22 homolog (MPXV 197) was severely attenuated in its pathogenesis, both in viral replication over time as well as morbidity and mortality^15^. These findings as well as the large size and high conservation of B22 family members strongly imply a major and indispensable role in OPXV biology. In line with this, studies using ectromelia (ECTV) infection in mice found that the insertion of a stop codon before its B22 ORF (corresponding protein termed “C15” in ECTV) largely prevents mortality in BALB/c mice^16^, which are highly susceptible to wild-type (WT) ECTV infection^21^. Together, these studies demonstrated conserved and prominent contributions to virulence by B22 proteins in diverse mammalian hosts and highlight their strong candidacy as potential targets for anti-OPXV therapeutics. However, the immunological consequences of B22 protein expression *in vivo* have been poorly defined.

ECTV infection of mice exemplifies a natural host-pathogen relationship with notable similarities to the disease progression of smallpox^22^, and alongside the high conservation of its B22 protein with those of MPXV and variola^17^, this model offers a broadly relevant avenue to study the critical contributions of B22 proteins to OPXV virulence. Using ECTV, we previously demonstrated that C15 is capable of potently inhibiting mouse CD4+ T cells, but not CD8+ T cells, post-peptide loading onto antigen-presenting cells (APCs)^17^, in contrast to other B22 family members, which had been demonstrated to inhibit both CD4+ and CD8+ T cells^15^. Furthermore, preliminary *in vitro* findings suggested that C15 functions by inhibiting the formation of APC-T cell synapses^17^. Further complicating the picture is the discovery that C15 contributes to viral dissemination *in vivo* as early as two days post-infection in C57Bl/6 (B6) mice^16,19^, well before the establishment of an adaptive immune response. Whole lymph node imaging revealed that C15 inhibits natural killer (NK) cell engagement of infected cells, resulting in much greater viral replication and dissemination at early time points in WT ECTV infection compared to ECTV C15^19^. These findings place C15, and B22 proteins by extension, into an extremely limited category of viral virulence factors that can inhibit both the innate and adaptive immune system through the targeting of separate immune cell populations^23^. Nevertheless, the consequences of C15 on the development of antiviral T cell responses and the dynamic interplay between C15 and NK cells *in vivo* has not been explored. Furthermore, whether C15 is capable of unequivocally inhibiting CD8+ T cells, like other B22 proteins, has not been thoroughly investigated.

Here, using a novel *in vitro* expression system, we demonstrate that, indeed, C15 potently inhibits CD8+ T cells, uncovering a third inhibitory activity of this enigmatic virulence factor. Counterintuitively, during *in vivo* infection of C57Bl/6 (B6) mice, in the face of C15, WT ECTV induced larger and more functional CD8+ T cell responses compared to ECTV C15, with phenotypic and transcriptomic analyses indicating stronger TCR signaling in WT-derived CD8+ T cells. Experiments in *Batf3*^*−/−*^ mice identified cDC1-mediated cross-presentation as the primary host workaround to CD8+ T cell inhibition by C15, but CD4+ T cell responses were similarly enhanced with C15 expression. Considering previous findings that NK cells heavily restrict ECTV C15 replication early in the draining lymph node, we determined that antigen load differences, driven by C15-mediated antagonism of NK cells, were the defining factors in the T cell responses to WT and ECTV C15. Using single cell RNA sequencing, we identified a candidate signaling scaffold that may be upstream of C15-mediated NK cell inhibition. Finally, we demonstrated that in BALB/c mice, which bear deficiencies in NK cell responses against ECTV, C15 more prominently targets CD4+ and CD8+ T cell responses, establishing the principle of conditional immune antagonism by C15.

## Results:

### The ECTV C15 protein inhibits both CD4+ and CD8+ T cells

**Figure 1.**
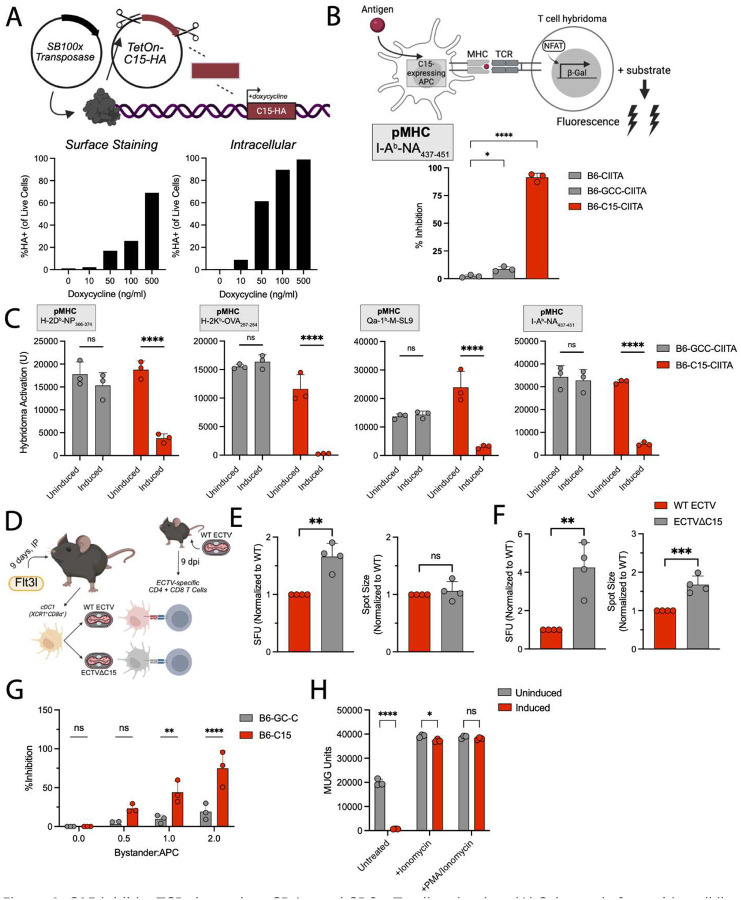
C15 inhibits TCR-dependent CD4+ and CD8+ T cell activation. (A) Schematic for stable cell line generation and flow cytometry data for surface and intracellular HAtag expression following induction. (B) Schematic for T cell hybridoma assay and % inhibition (mean SD) of indicated hybrid with inducible cell lines. (C) Hybridoma activation (mean SD) with inducible cell lines across various T cell hybridomas, as indicated. (D) Schematic for E-F. (E and F) Spot-forming unit (SFU) counts and spot size (mean SD) from WT- or C15-infected cDC1 co-cultures with ECTV-specific CD8+ (E) or CD4+ (F) T cells. G) % inhibition (mean SD) of I-A^b^-NA_437–451_-specific T cell hybrid with various ratios of induced MHCII-negative bystanders to MHCII+ APCs. H) Hybridoma activation (mean SD) by the inducible C15 cell line with or without PMA/ionomycin. (B and C) Representative of three experiments. (E-F) Data shown are pooled from four separate infections. (G) Dot represents individual experiment. (B) One-way ANOVA with Dunnett correction. (C, G, H) Two-way ANOVA with Sidak correction. (E and F) Unpaired t-test. Related to Figure S1.

To evaluate the breadth of T cell inhibition by C15, we established an inducible expression system that utilizes the Sleeping Beauty transposase to deliver a doxycycline-controlled C15-(HA tag) gene cassette into C57Bl/6 (B6)-derived skin fibroblasts (B6-C15) (Figure 1A)^24^. We validated the expression and membrane trafficking of C15 via flow cytometry, demonstrating dose-dependent increases in HA-tag+ cells (Figure 1A). In previous work, we demonstrated that C15 inhibits CD4+ T cell activation^17^. To confirm this activity in our inducible expression system, we transduced B6-C15 cells with lentivirus encoding the class II transactivator (CIITA) (Figure S1A), induced overnight, and performed a T cell hybridoma assay (Figure 1B)^25^. As controls, we included an uninduced condition and an inducible cell line expressing the comparably large transmembrane protein guanylyl cyclase C (GC-C) (Figure S1B)^26^. In line with our previous findings, C15 expression significantly reduced activation of MHCII-restricted T cell hybridomas (Figure 1B).

Initial publications on B22 family proteins reported that these proteins inhibit both CD4+ and CD8+ T cell activation^15^. In contrast, we previously demonstrated that C15 inhibits only CD4+ T cells^17^. We speculated this selective effect was due to low C15 expression levels in our previous systems, and, indeed, this is supported by the demonstration that inhibition of CD4+ T cell hybridomas by C15 is reduced when expression is driven by a weaker promoter (UbC) (Figure S1C). We investigated the inhibition of various CD8+ T cell hybridomas using our inducible cell line, finding that C15 inhibited all CD8+ T cell hybridomas similarly to CD4+ T cell hybridomas (Figure 1C). To ensure inhibition was not an artifact of our *in vitro* system, we infected FMS-like tyrosine kinase 3 ligand (Flt3l)-derived type 1 conventional dendritic cells (cDC1s) (Figures S1D–G) with ECTV-eGFP (WT)^27^ or ECTV C15 (C15)^17^ (Figure S1H–I) for co-culture with ECTV-specific primary CD8+ or CD4+ T cells (Figure 1D). Restimulation of CD8+ (Figure 1E) and CD4+ (Figure 1F) T cells by C15-infected cDC1s was significantly greater compared to WT-infected cDC1s, although the relative inhibition was much more potent in the CD4+ T cell co-culture. Furthermore, CD4+ T cell IFN spots were significantly larger when co-cultured with C15-infected cDC1s compared to WT-infected cDC1s (Figure 1F).

C15-mediated inhibition was not due to downregulation of any MHC or co-stimulatory molecule (Figure S1J), suggesting that the mechanism of C15 inhibition is either MHC-independent or fundamental to all antigen-specific APC-T cell interactions. Previous data indicated that C15 functions post-peptide loading^17^, but it has not yet been shown whether C15 modulates the number of peptide-MHC (pMHC) complexes on the cell surface. Using TCR-like antibodies that recognize either pMHCI (H-2K^b^-SIINFEKL) or pMHCII (I-A^b^-Ea_52–68_) complexes, we found that C15 does not alter the abundance of pMHC complexes on the cell surface, derived from either mRNA lipid nanoparticle-encoded protein (Figures S1K–M) or pulsed peptide (Figures S1N–O). Taken together, our data indicate that, in addition to its impact on NK cells, C15 can inhibit CD4+ and CD8+ T cells without disrupting pMHC complex presentation at the cell surface.

To gain additional insights into C15 inhibitory activity, we asked whether C15 could inhibit T cell hybridoma activation from a “bystander cell”. To accomplish this, we induced MHCII-negative C15-HA-tag cells (bystander) and co-incubated them with MHCII+ B6 fibroblasts (APCs) in the presence CD4+ T cell hybridomas and exogenous peptide. Here, C15 inhibited hybridoma activation in a dose-dependent manner (Figure 1G), but this was not mediated by a secreted fragment since supernatant from high density cell cultures did not have an inhibitory effect (Figure S1P). To ask whether C15 antagonizes TCR-independent calcium flux or intracellular signaling, we treated hybridomas with ionomycin and phorbol 12-myristate 13-acetate (PMA). In this context, C15 was unable to inhibit hybridoma activation (Figure 1H), suggesting TCR-dependent inhibition like other B22 protein members^15^. Thus, our data indicate that C15 is cell-associated and can inhibit TCR-dependent CD4+ and CD8+ T cell activation without necessarily being expressed by the APC.

### CD8+ T cell responses are enhanced in the face of C15

**Figure 2.**
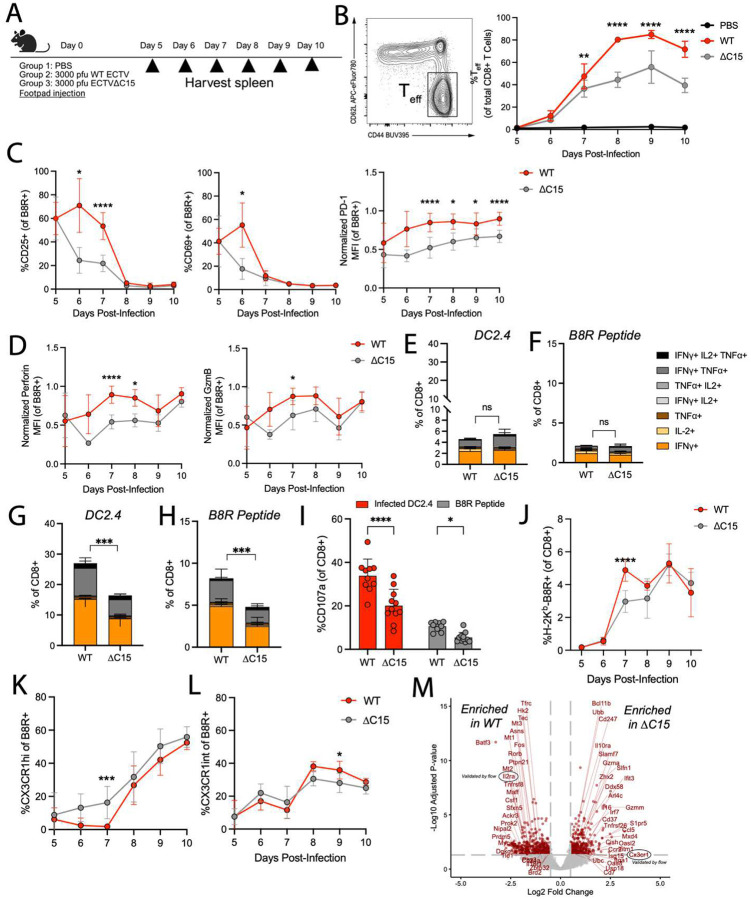
C15 expression promotes enhanced CD8+ T_eff_ responses. (A) Schematic for B-D and J-L. (B) Representative flow gating for CD8+ T_eff_ and the proportion of CD8+ T_eff_ (of total CD8+ T) (mean SD) over time in the spleen for indicated infection conditions. (C) %+ (CD25 or CD69) or normalized MFI (PD-1) of B8R+ CD8+ T_eff_ (mean SD) over time for WT or C15 infection. (D) Normalized MFI (perforin or GzmB) of B8R+ CD8+ T_eff_ (mean SD) over time for WT or C15 infection. (E-H) % cytokine-producing CD8+ T cells (mean SD) following restimulation of splenocytes with infected DC2.4 cells (E/G) or peptide pulsing with B8R peptide (F/H) at 7 (E/F) or 9 (G/H) dpi. (I) %CD107+ CD8+ T cells (mean SD) following restimulation of splenocytes 9 dpi as indicated. (J) Proportion of B8R+ CD8+ (mean SD) over time for WT or C15 infection. (K/L) Quantification of CX3CR1hi (K) and CX3CR1int (L) B8R+ CD8+ T_eff_ (mean SD) over time in WT- or C15-infected mice. (M) DEGs in CD8+ T_eff_ between WT and C15 infection. (B-D, J-L) Data pooled across 1–3 independent experiments with n=4–15 for each group. (E-F) Data derived from 4–5 mice per group. (G-I) Data pooled across two independent experiments with n=10–11 per group. (I) Dot represents one mouse. (M) Sequencing data derived from n=4–5 per group. (B-D, J-L) Mixed-effects model with Sidak correction. (E-H) Unpaired t-test. (I) Two-way ANOVA with Fisher’s LSD test. Related to Figure S2.

Given the importance of CD8+ T cells in protective immunity against ECTV^28,29^, we footpad infected B6 mice with either WT or C15 and evaluated splenic CD8+ T cell responses (Figure 2A). Contrary to expectations, CD8+ effector T cell (CD8+ T_eff_) responses to infection were significantly greater as a percentage of total CD8+ T cells during WT infection, with differences being apparent as early as 7 days post-infection (dpi) (Figure 2B). To probe antigen-specific CD8+ T_eff_, we stained splenic CD8+ T cells with an H-2K^b^ tetramer loaded with the immunodominant B8R_20–27_ epitope (B8R+) (Figure S2A). Notably, at 6 dpi, WT-derived B8R+ T_eff_ had greater expression of the activating markers CD69 and CD25, which persisted through 7 dpi (Figure 2C). Furthermore, PD-1, perforin, and granzyme B (GzmB) were upregulated starting at 7 dpi, and PD-1 expression was maintained through 10 dpi (Figures 2C–D). Together, these data suggest that WT infection (C15-expressing), counterintuitively, induced a larger and more activated CD8+ T cell response to infection compared to C15.

To further investigate whether CD8+ T cell responses are, in fact, enhanced in the face of C15, we assessed cytokine production by and cytolytic potential of CD8+ T cells from either WT or C15-infected mice at 7 or 9 dpi. This was done via restimulation of splenocytes with C15-infected DC2.4 cells or B8R peptide (Figure S2B). While there were no significant differences in the proportion of cytokine-producing CD8+ T cells with either restimulation condition at 7 dpi (Figures 2E–F), by 9 dpi, a substantially greater proportion of WT-derived CD8+ T cells were cytokine-producing and exhibited higher cytotoxic potential compared to C15-infected mice from both restimulation conditions (Figures 2G–I). The greater functionality of WT-derived total CD8+ T cells can be attributed to the larger population of CD8+ T_eff_ in WT-infected mice at 9 dpi (Figure 2B), but the lower functionality of B8R peptide-stimulated T cells in C15-infected mice indicate less cytokine-producing capacity on a per cell basis, as the proportion of B8R+ CD8+ T cells is equivalent between WT and C15 at this timepoint (Figure 2J). In line with decreased functionality, a significantly greater proportion of C15-derived B8R+ T_eff_ highly expressed CX3CR1 (CX3CR1^hi^) (Figures 2K and S2C–D), a marker of terminal differentiation that has been negatively associated with polyfunctionality^30,31^. Furthermore, at 9 dpi, there were more CX3CR1^int^ B8R+ T_eff_ in WT-infected mice compared to in C15-infected mice (Figure 2L). Together, these data indicate that B8R+ T_eff_ in WT-infected mice are more polyfunctional and less terminally differentiated than those from C15 infection.

Lastly, to couple phenotypic and functional differences with transcriptional changes, we performed bulk RNA-sequencing on splenic CD8+ T_eff_ at 7 and 10 dpi (Figures 2M and S2E and Table S1). At 7 dpi, differentially expressed genes (DEGs) within CD8+ T_eff_ from WT-infected mice were enriched for targets of MYC – a prominent regulator downstream of T cell activation (Figure S2F)^32^, IL-2 signaling (Figure S2G), and zinc ion binding (Figure S2H), which has been positively associated with TCR engagement^33,34^. Gene ontology (GO) analysis further demonstrated enrichment in genes related to mRNA processing, RNA localization, ribosome biogenesis, and DNA metabolism (Figure S2I). In contrast, C15-derived CD8+ T_eff_ were strongly enriched for genes downstream of type I interferon (IFN) (Figure S2J), genes associated with an NK cell-like phenotype (Figure S2K), and genes implicated in negative regulation of TCR signaling (*Cd37*, *Cish*, *Klr1g*, *Nrp2*, *Ptpn6*, *Tnk2*)^35–40^. At 10 dpi, WT-derived CD8+ T_eff_ maintained greater expression of several AP-1-associated factors (*Fos*, *Jun*, *Egr1*)^41,42^ while C15-derived CD8+ T_eff_ had greater expression of *Bach2* and *Zbtb20*, which restrain chromatin accessibility of AP-1 factors^43,44^ and limit the metabolic capacity of CD8+ T cells^45^, indicating the persistence of T cell phenotypes from earlier infection timepoints (Figures S2L–M and Table S1). These transcriptional data support the findings that WT infection drives larger and functionally enhanced CD8+ T_eff_ compared to C15-derived CD8+ T_eff_, in line with our direct assessments of CD8+ T cell phenotype.

### CD8+ T cells have higher avidity during WT infection

**Figure 3.**
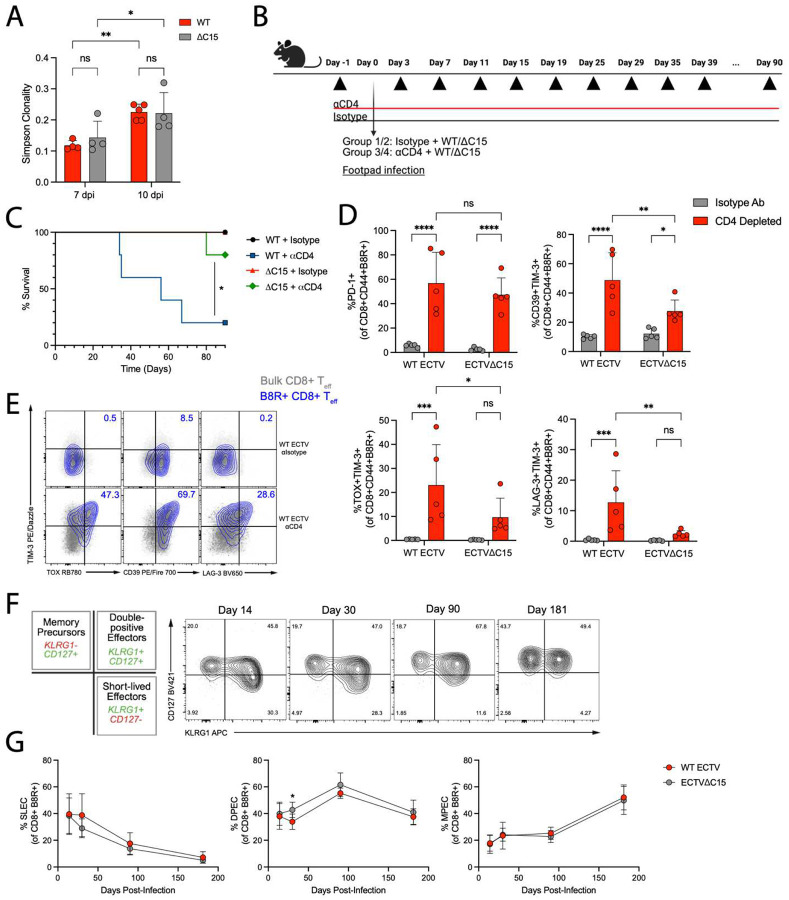
CD8+ T cell clonality, avidity and memory following WT or C15 infection. (A) Simpson clonality (mean SD) of B8R+ CD8+ T_eff_ at indicated dpi for WT or C15. (B) Schematic for C-E. (C) Survival curve for mice infected with WT or C15 either untreated or depleted of CD4+ T cells. (D) % PD-1+, CD39/TIM-3+, TOX/TIM-3+, or LAG-3/TIM-3+ of B8R+ CD8+ T_eff_ (mean SD) at 30 dpi with WT or C15 either untreated or depleted of CD4+ T cells. (E) Representative flow cytometry plots for values quantified in (D). (F) Markers of memory populations and representative flow cytometry plots from each key timepoint. (G) %SLEC, DPEC, or MPEC (mean SD) of B8R+ CD8+ T cells over time in WT- or C15-infected mice. (A, D) Dot represents individual mouse. (G) Data derived from 5 mice per group. (A) Two-way ANOVA with Fisher’s LSD test. (C) Mantel-Cox test. (D) Two-way ANOVA with Tukey correction. (G) Unpaired t-test for each timepoint. Related to Figure S3.

We speculated that WT infection induced larger CD8+ T_eff_ responses than C15 infection due to broader T cell priming from a more diverse array of epitopes. Consistent with this, by 10 dpi, there were significantly less B8R+ T cells among T_eff_ during WT infection compared to C15 (Figure S3A). This suggests that antigen-specific responses during WT infection are more antigenically diverse and less dominated by the B8R epitope than those during C15 infection. Conversely, a significantly greater proportion of B8R+ T_eff_ during C15 infection suggests that there may be differences in clonal diversity among B8R+ CD8+ T cells. To investigate this, we performed bulk TCR sequencing on B8R+ T_eff_ cells from WT- or C15-infected mice at 7 and 10 dpi (Figure S3B). However, there were no differences in clonal diversity between WT- or C15-infected mice at either timepoint (Figure 3A). Furthermore, there were not major differences in either V or J gene usage between infection conditions (Figures S3C–D). Therefore, C15 expression does not alter the TCR repertoire of B8R+ T cells, and there is no evidence for selective clonal expansion in either infection condition.

An alternative explanation for the enhanced CD8+ T_eff_ responses during WT infection is greater T cell avidity, as higher avidity T cells have been shown to proliferate more efficiently *in vivo*^46^. Therefore, we tested the relative avidity of B8R+ CD8+ T_eff_ from WT- or C15-infected mice using a tetramer decay assay (Figure S3E)^47^. We found no differences in the rate of tetramer decay between infection conditions, suggesting similar avidities (Figure S3F), but the B8R tetramer-TCR affinity may be too high for a meaningful investigation of T cell avidity. As an orthogonal approach, we evaluated the phenotype of WT- or C15-derived CD8+ T_eff_ over time in a model of chronic antigen stimulation, as high avidity T cells have been shown to undergo greater exhaustion in these contexts^47–50^. To generate a model of chronic antigen stimulation during ECTV infection, we depleted B6 mice of CD4+ T cells prior to infection with WT and C15 (Figure 3B), as previous work suggested CD4+ T cells are required to promote efficient CD8+ T cell contraction post-infection^28^. WT-infected mice began succumbing to infection by approximately 35 dpi, with 80% deceased by 70 dpi, whereas C15-infected mice largely survived throughout this timeline (80% survival) (Figure 3C). By 30 dpi, CD8+ T_eff_ in CD4-depleted mice generally had elevated PD-1 expression, but WT-derived CD8+ T_eff_ exhibited higher expression of the terminal exhaustion markers CD39, TOX, and LAG-3 compared to those from C15-infected mice (Figure 3D)^51–53^, which were enriched within B8R+ T cells (Figure 3E). CD8+ T cell exhaustion was highly progressive, with systemic upregulation of terminal exhaustion markers in the days prior to death (Figure S3G). Together, these findings suggest that WT-derived CD8+ T_eff_ have greater avidity that drives enhanced proliferation and functionality *in vivo*.

### CD8+ T cells exhibit similar memory trajectories during WT and C15 infection

Greater avidity of CD8+ T_eff_ during WT infection suggests that CD8+ T cell memory may be enhanced compared to C15 infection^54^. To investigate this, we tracked B8R+ T cells over the course of 6 months post-WT or - C15 infection and monitored short-lived effector cells (SLEC), memory precursor effector cells (MPEC), and “double positive” (KLRG1+ CD127+) effector cells (DPEC) (Figure 3F), which are thought to be the primary source of “exKLRG1” long-lived memory cells^55–57^. SLEC and DPEC were the dominant populations at 14 dpi in all mice (Figure 3G), while DPEC and MPEC were dominant at 181 dpi, with roughly equal proportions. Notably, the trajectories of memory development were similar following WT and C15 infection (Figure 3G). Therefore, despite C15 expression substantially enhancing the magnitude and function of the acute CD8+ T cell response, there does not appear to be long-term effects on the development of CD8+ T cell memory against the B8R epitope.

### Cross-presentation compensates for C15-mediated inhibition of direct presentation to CD8+ T cells

**Figure 4.**
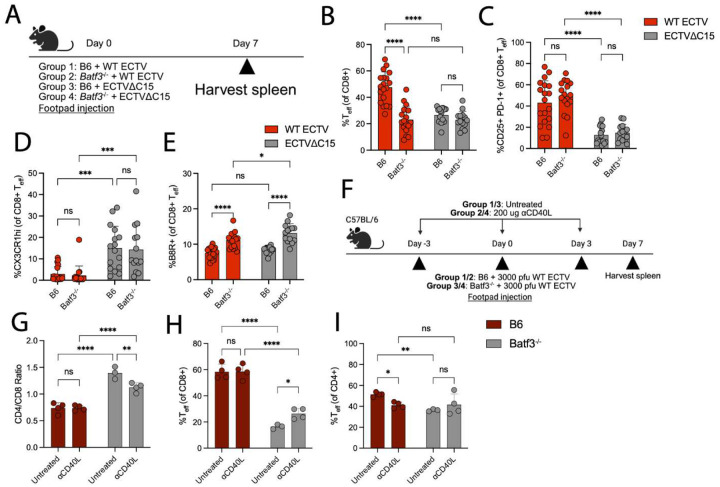
Cross-presentation compensates for inhibition of CD8+ T cells by C15. (A) Schematic for C-L. (B-E) %T_eff_ of total splenic CD8+ T cells (B), %PD-1+CD25+ of CD8+ T_eff_ (C), %CX3CR1hi of CD8+ T_eff_ (D), %B8R+ of CD8+ T_eff_ (E) (mean SD) at 7 dpi with WT or C15 in B6 or *Batf3*^*−/−*^ mice. (F) Schematic for G-I. (G) CD4/CD8 ratio of CD3+ splenocytes (G), %T_eff_ of splenic CD8+ T cells (H), and %T_eff_ of splenicCD4+ T cells (mean SD) from WT-infected B6 or *Batf3*^−/−^ mice untreated or treated with CD40L blocking antibody 7 dpi. (B-E) Data pooled across 3 independent experiments. (B-E, G-I) Dot represents one mouse. (B-E) Two-way ANOVA with Tukey correction. (G-I) Two-way ANOVA with Fisher’s LSD test. Related to Figure S4.

The juxtaposition of potent CD8+ T cell inhibition by C15 *in vitro* and the greater CD8+ T cell responses during WT infection suggests compensatory mechanisms *in vivo*. While C15 can effectively block antigen presentation from a bystander cell (Figure 1G), we speculated that cross-presentation drives enhanced CD8+ T cell responses during WT infection compared to C15. Therefore, we used the *Batf3*^*−/−*^ mouse model, which is deficient in the development of CD8 + cDC1s – the primary mediators of cross-presentation during poxviral infections^58,59^ – while allowing for ECTV viral replication^60^. We infected either B6 or *Batf3*^*−/−*^ mice with WT or C15 and investigated their splenic CD8+ T cell responses at 7 dpi (Figure 4A). Similar to other reports^61^, we found that the ratio of splenic CD4+/CD8+ T cells 7 dpi was significantly higher in *Batf3*^*−/−*^ mice compared to B6 counterparts, highlighting an intrinsic deficit in CD8+ T cell proliferation independent of infection condition (Figure S4A). However, the development of CD8+ T_eff_ was significantly reduced in WT-infected *Batf3*^*−/−*^ mice compared to their B6 counterparts, while CD8+ T_eff_ were unchanged in C15-infected mice (Figure 4B). While CD8+ T_eff_ did not differ in their expression of CD25 or CX3CR1 between B6 and *Batf3*^*−/−*^ mice (Figures 4C–D), the proportion of B8R+ T_eff_ was significantly upregulated in *Batf3*^*−/−*^ mice independent of infection condition (Figure 4E), suggesting that B8R is more abundantly presented when cross-presentation is stunted. CD8+ T_eff_ also trended towards or exhibited greater expression of TIM-3 in *Batf3*^*−/−*^ mice (Figure S4B), which may explain the lower capacity for *Batf3*^*−/−*^ CD8+ T_eff_ to proliferate^62^.

CD40-CD40L signaling between cDC1s and CD4+ T cells has been suggested as critical in the “licensing” of cDC1s for CD8+ T cell priming^63^. To exclude the possibility that the differences observed with WT and *Batf3*^*−/−*^ mice could be explained by differences in CD40-CD40L signaling, we treated B6 and *Batf3*^*−/−*^ mice with CD40L blocking antibody during WT infection (Figure 4F). CD70 expression, a response downstream of CD40 ligation^64^, on cDC1s was downregulated in B6 mice treated with CD40L, indicating successful blockade (Figures S4C–D). Batf3-independent cDC1s responded with limited CD70 upregulation but greater overall CD40 expression (Figures S4E), and CD40L treatment expanded cDC1s in *Batf3*^*−/−*^ mice (Figure S4F). CD40L blockade did not dampen CD8+ T_eff_ responses to WT infection in B6 mice, but it increased the induction of CD8+ T_eff_ in *Batf3*^*−/−*^ mice (Figures 4G–H), implicating non-canonical CD40L signaling in negatively regulating CD8+ T cell responses in *Batf3*^*−/−*^ mice during ECTV infection^65,66^. In B6 mice, there was a decrease in CD4+ T_eff_ following CD40L treatment (Figures 4I), which was expected given the role for CD40L in activation of CD4+ T_eff_^67,68^. Together, these data demonstrate that phenotypes in WT-infected *Batf3*^*−/−*^ mice are not due to a lack of CD40-CD40L engagement, and that T cell responses during WT infection are more sensitive to deficits in cross-presentation compared to C15 infection, in line with inhibition of direct presentation to CD8+ T cells by C15.

### CD4+ T cell responses are similarly enhanced during WT infection compared to C15

**Figure 5.**
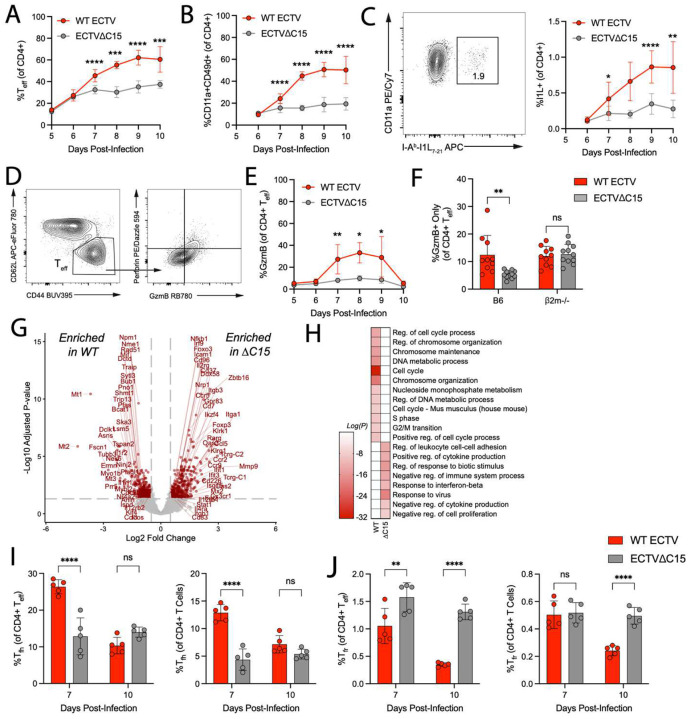
CD4 T_eff_, CD4-CTL and T_fh_ responses are enhanced during WT infection. (A-B) %T_eff_ (A) and CD11a+CD49d+ (B) (mean SD) of total splenic CD4+ T cells in WT- or C15-infected mice. (C) Representative flow plot of I1L+ CD4+ T cells and quantification of splenic I1L+ CD4+ T cells (mean SD) over time in WT- or C15-infected mice. (D) Representative flow cytometry gating for the identification of CD4-CTLs. (E) %GzmB+ of CD4+ T_eff_ (mean SD) over time in WT- or C15-infected mice. (F) %GzmB+ perforin+ (mean SD) of CD4+ T_eff_ for each infection condition in indicated mouse strains. (G) DEGs in CD4+ T_eff_ between WT and C15 infection. (H) Enriched GO terms in CD8+ T_eff_ from each infection condition. (I-J) %T_fh_ (I) of %T_fr_ (J) (mean SD) of total splenic CD4+ T cells or CD4+ T_eff_ at 7 and 10 dpi in different infection conditions. (A-C, E) Data pooled across 1–3 independent experiments with n=4–15 for each group. (F, I-J) Dot represents individual mouse. (G-H) Sequencing data derived from n=5 for each group. (A-C, E) Mixed-effects model with Sidak correction. (F, I-J) Two-way ANOVA with Sidak correction. Related to Figure S5.

Inhibition of CD4+ T cells by C15 has been previously reported *in vitro* and *ex vivo*^17^. Furthermore, here, C15 demonstrated enhanced potency against CD4+ T cells *ex vivo* compared to CD8+ T cells (Figure 1F). We therefore speculated that CD4+ T cell responses may be more sensitive to C15 inhibition *in vivo*. However, like CD8+ T cells, we found that the induction of CD4+ T_eff_ (CD62L-CD44+) was greater during WT infection compared to C15 (Figure 5A). Furthermore, the development of antigen-experienced CD4+ T cells (CD11a+CD49d+)^69,70^, which correlate with CD4+ T_eff_ (Figure S5A), and antigen-specific (I-A^b^-I1L_7–21_-specific, I1L+)^69^ CD4+ T cells was greater in spleens of WT-infected mice by 7 dpi (Figures 5B–C), without an impact to immunodominance over time (Figure S5B).

A hallmark of ECTV infection is the development of cytotoxic CD4+ T cells (CD4-CTLs, GzmB+ and/or perforin+)^69,71^. As previously described^69,71^, WT infection induced a substantial population of CD4-CTLs (Figures 5D–E). In contrast, there was little to no CD4-CTL induction during C15 infection (Figure 5E). To determine whether CD4-CTL induction is strictly dependent on C15, we compared CD4-CTL development during C15 infection of 2m^−/−^ mice, as the absence of CD8+ T cells in these mice (Figure S5C) induces a compensatory increase in CD4-CTLs^72^. Notably, the induction of CD4-CTLs was rescued in 2m^−/−^ mice following C15 infection (Figure 5F), suggesting that CD4-CTL responses are not intrinsically linked to C15 expression.

To gain broader insights into the impact of C15 on CD4+ T_eff_ cell responses, we performed bulk RNA-sequencing on sorted splenic CD4+ T_eff_ 7 dpi (Figures 5G, S5D and Table S2). WT-derived CD4+ T_eff_, like CD8+ T_eff_, had increased expression of transcriptional modules downstream of IL-2 signaling (Figure S5E) and MYC expression (Figure S5F), as well as upregulation of genes related to oxidative phosphorylation (Figure S5G) and the unfolded protein response (Figure S5H). GO term enrichment further identified an upregulation of genes associated with the cell cycle in WT-derived CD4+ T_eff_ (Figure 5H). The transcriptional phenotype of CD4+ T_eff_ during C15 infection was similar to that of CD8+ T_eff_ (Figures 5G and S5I–J), but there was also greater expression of regulatory T cells (T_reg_)-associated genes (*Runx1, Foxo1, Foxo3, Foxp3, Gpr83, Ikzf4, Nrp1*)^73–77^ (Figure 5G), suggesting that C15-derived CD4+ T_eff_ are more skewed towards immunosuppressive phenotypes.

Lastly, we asked whether CD4+ T follicular helper cell (T_fh_) responses recapitulated our findings in CD4+ T_eff_, examining the balance of T_fh_ and CD4+ T follicular regulatory cells (T_fr_), as antibodies are a critical determinant of protection against ECTV^28^. We measured the induction of T_fh_ and T_fr_ cells in the spleen and inguinal lymph node (iLN) at 7 and 10 dpi (Figure S5K). WT-infected mice had a significantly greater proportion of T_fh_ within both splenic CD4+ T_eff_ and total CD4+ T cells 7 dpi, but this difference was not apparent at 10 dpi (Figure 5I). In contrast, C15-infected mice had greater proportions of T_fr_ in splenic CD4+ T_eff_ at 7 and 10 dpi and within total CD4+ T cells 10 dpi (Figure 5J). Between infection conditions, we found no significant differences in the induction of T_fh_ or T_fr_ cells within the iLN (Figures S5L–M). Notably, these differences in T_fh_ did not change anti-ECTV IgG antibody titers 28 dpi (Figure S5N). Overall, as with CD8+ T cell responses, CD4+ T cell responses to ECTV infection are enhanced in the presence of C15 – at odds with the potent inhibition observed *in vitro* and *ex vivo* (Figure 1).

### Cross-protective immunity is maintained in the absence of C15

Given WT- and C15-infected mice displayed similar CD8+ T cell memory trajectories and equivalent anti-ECTV IgG development, we asked whether significant deficits in acute T cell responses during C15 infection impacted the establishment of cross-protective immunity. Therefore, we challenged WT- or C15-immunized mice 30 dpi with a lethal dose of the related orthopoxvirus, cowpox (CPXV) (Figure S5O). All ECTV-immunized (WT or C15) mice survived (Figure S5P), with increases in the CD8+ T_eff_ population following challenge (Figure S5Q). Together, these data demonstrate that the transcriptional, phenotypic and functional differences in CD8+ and CD4+ responses during WT or C15 infection, under the specified conditions, do not hinder the establishment of cross-protective immunity.

### T cell differences between WT and C15 infection are driven by antigen load

While cDC1-mediated cross-presentation explains the enhanced CD8+ T cell responses in the face of C15 expression, this mechanism does not explain the deficiencies in CD8+ and CD4+ T cell responses during C15 infection, which should be significantly enhanced compared to WT according to *in vitro* and *ex vivo* findings (Figure 1)^17^. We next considered the possibility that the interaction between C15 and its third cellular target, NK cells, impacts T cell responses. We have previously demonstrated that C15 replication in B6 mice is heavily restricted in the draining lymph node due to NK cell-mediated control, which is effectively antagonized by C15^19^. Therefore, we hypothesized that decreased antigen load during C15 infection may underlie the differences in T cell responses described above, and this notion is further supported by the association between CD4-CTL induction and viral replication^71^, both of which are markedly reduced during C15 infection.

**Figure 6.**
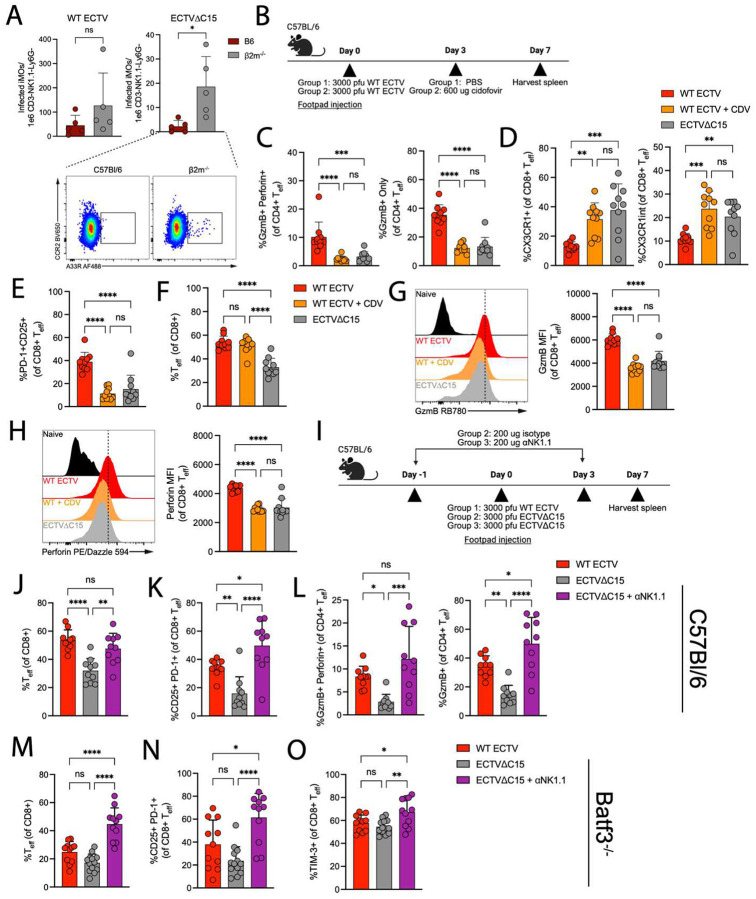
NK cells restrict antigen load during C15 infection to dampen T cell responses. (A) #Infected iMOs per 1 × 10^6^ CD3-NK1.1-Ly6G- splenocytes (mean SD) 7 dpi with WT or C15 in each corresponding mouse strain, with representative flow cytometry plots below. (B) Schematic for C-H. (C-F) %GzmB+perforin+ and GzmB+ only of CD4+ T_eff_ (C), %CX3CR1+ and CX3CR1int of CD8+ T_eff_ (D), %PD-1+CD25+ of CD8+ T_eff_ (E), and %T_eff_ of total splenic CD8+ T cells (F) (mean SD) at 7 dpi across infection conditions. (G-H) Representative histograms and quantified MFI of GzmB (G) and perforin (H) for CD8+ T_eff_ (mean SD) at 7 dpi across infection conditions. (I) Schematic for J-L. (J-L) %T_eff_ of total CD8+ T cells (J), %PD-1+CD25+ of CD8+ T_eff_ (K), and %GzmB+perforin+ and GzmB+ only of CD4+ T_eff_ (L) (mean SD) at 7 dpi across infection conditions. (M-O) %T_eff_ of total splenic CD8+ T cells (M), %PD-1+CD25+ of CD8+ T_eff_ (N), %TIM-3 of CD8+ T_eff_ (O) (mean SD) at 7 dpi across infection conditions in B6 or *Batf3*^*−/−*^ mice. (C-H, J-O) Data pooled across 2 independent experiments. (A, C-H, J-O) Each dot represents one mouse. (A) Unpaired t-test. (C-H, J-O) Ordinary one-way ANOVA with Tukey correction. Related to Figure S6.

The finding that C15 infection of 2m^−/−^ mice rescued the induction of CD4-CTLs (Figure 5F) implies that C15 infection replication is enhanced in the absence of a functional CD8+ T cell compartment. Indeed, B6 mice pretreated with anti-CD8 depleting antibody succumbed to both WT and C15 infection (Figure S6A). To directly quantify viral burden in 2m^−/−^ mice *ex vivo*, we used an anti-MPXV A35R (A33R in ECTV) monoclonal antibody^78^, which displayed strong cross-reactivity with ECTV (Figures S6B–C). Notably, inflammatory monocytes (iMOs, Figure S6D) from C15-infected 2m^−/−^ mice were more highly infected than their B6 counterparts (Figure 6A). Thus, differences in viral load may explain the impairment of T cell responses during C15 infection.

To explicitly test the relationship between antigen load and T cell phenotype, we first infected B6 mice with WT ECTV and treated with cidofovir (CDV) 3 dpi to limit late viral replication (Figure 6B)^79,80^. In line with previous reports^71^, CDV treatment significantly dampened the induction of CD4-CTLs (Figure 6C). Furthermore, CDV treatment also increased the proportion of CX3CR1+ and CX3CR1^int^ CD8+ T_eff_ (Figure 6D) and reduced PD-1+ CD25+ CD8+ T_eff_ (Figure 6E). CDV treatment did not impact the magnitude of CD8+ T_eff_ generation among total CD8+ T cells (Figure 6F), but the expression of granzyme B and perforin was reduced to a level equal to C15 infection (Figures 6G–H). To directly implicate NK cells as the mediator of key antigen restrictions during C15 infection, we compared T cell responses to WT or C15 infection in untreated mice with that of NK-depleted, C15-infected mice (Figure 6I). NK cell depletion restored CD8+ T_eff_ induction to levels equivalent to WT infection (Figure 6J) and increased CD25 and PD-1 expression above that of WT infection (Figure 6K). Furthermore, NK cell depletion rescued CD4-CTL induction (Figure 6L). Together, these data demonstrate that NK cell-infected cell interactions and their subsequent impact on antigen load are the defining factors in the CD4+ and CD8+ T cell response to WT and C15 infection in B6 mice.

As a proof-of-principle, we revisited C15 infection of *Batf3*^*−/−*^ mice, where the proportion of CD8+ T cells that developed into T_eff_ by 7 dpi was equivalent to B6 counterparts (Figure 4B). In this model, we speculated that lower antigen load in combination with deficiencies in cross-presentation synergize to ablate CD8+ T_eff_ expansion during C15 infection in *Batf3*^*−/−*^ mice, and this notion is supported by the finding that the proportion of CD8+ T_eff_ at 9 dpi is unchanged during C15 infection compared to WT infection, which underwent a modest expansion (Figures S6E).

Given findings thus far, we reasoned that increases in antigen load during C15 infection of *Batf3*^*−/−*^ mice should propel CD8+ T_eff_ expansion beyond that of WT infection, as direct presentation is more efficient than cross-presentation^81^. We depleted NK cells in *Batf3*^*−/−*^ mice prior to C15 infection, and in line with our hypothesis, NK cell depletion pushed CD8+ T_eff_ expansion beyond that of WT-infected *Batf3*^*−/−*^ mice (Figure 6M) and enhanced CD25 (Figure 6N) and TIM-3 expression (Figure 6O). Thus, restoration of antigen load during C15 infection of *Batf3*^*−/−*^ mice, which has unobstructed direct priming due to the absence of C15, led to enhanced CD8+ T_eff_ responses compared to WT infection. This further supports early antigen load restriction of C15 by NK cells as the critical bottleneck in the establishment of robust antiviral T cell responses.

### NK and T cells undergo distinct transcriptional changes in the dLN during C15 infection

**Figure 7.**
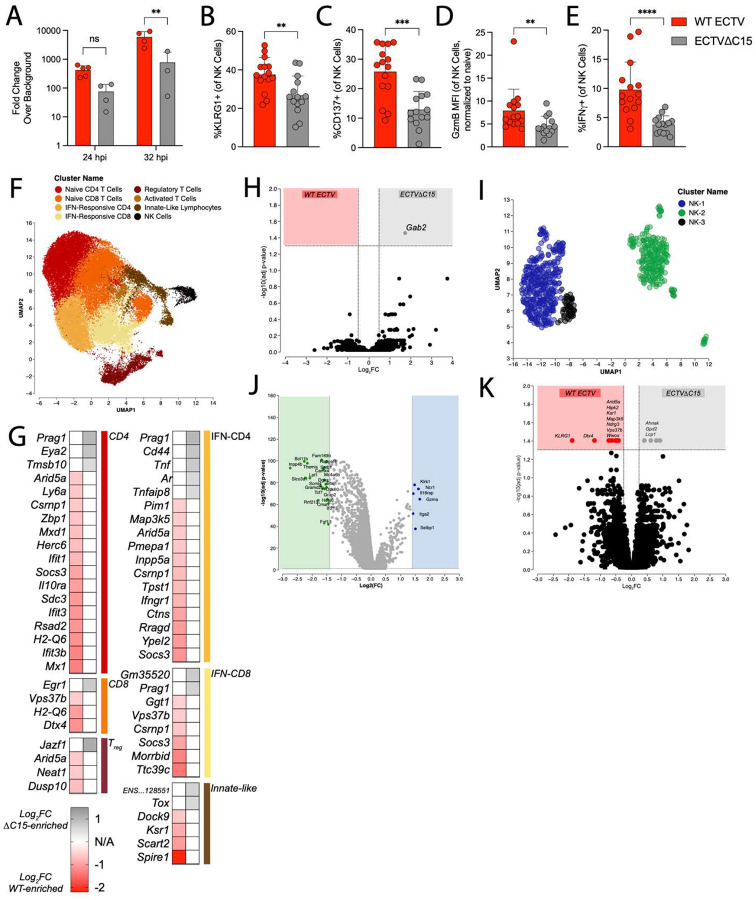
NK cells undergo distinct transcriptional changes during C15 infection. (A) Fold change of *EVM003* expression (mean SD) in WT- or C15-infected dLNs at 24 or 32 hpi. (B-E) %KLRG1+ (B), %CD137+ (C), normalized GzmB MFI (D), and %IFN + (E) (mean SD) of NK cells in WT or C15-infected dLNs at 48 hpi. (F) UMAP of scRNA-seq data from dLN 24 or 48 hpi with WT or C15. (G) Heatmap of DEGs across Leiden clusters at 48 hpi with WT or C15. (H) Volcano plot of DEGs in NK cells 24 hpi with WT or C15. (I) UMAP of subset NK cell clusters. (J) Volcano plot of DEGs between NK-1 and NK-2 subset clusters. (K) Volcano plot of DEGs in in the NK-2 sub-cluster 48 hpi with WT or C15. (A-E) Dot represents individual mouse. (B-E) Data pooled across 2–3 independent experiments. (F-K) Sequencing data derived from n=4–5 for each group. (A) Two-way ANOVA with Fisher’s LSD test. (B-E) Unpaired t-test. Related to Figure S7.

Early engagement and antagonism of NK cell-mediated control by C15 is critical to permit efficient viral replication in the dLN^19^, ultimately driving enhanced CD8+ and CD4+ T cell responses to infection. To investigate the mechanistic basis, we first assessed the transcriptional differences in the dLN during WT and C15 infection at 48 hpi, a time point prior to measurable differences in viral titer^19^. We performed bulk RNA sequencing on dLNs harvested 48 hpi (Figures S7A–B and Table S3). GO term enrichment of DEGs identified a handful of terms that were different between infection conditions (Figure S7C). Most notable terms were production of reactive oxygen species in WT-infected and macroautophagy in C15-infected dLNs. To more precisely identify transcriptional differences, we performed single cell RNA sequencing (scRNA-seq). To ensure we captured the earliest points of divergence between WT and C15 infection, we performed qPCR for viral RNA at 24 and 32 hpi as well as probed NK cell phenotypes at 48 hpi. There was significantly more viral RNA by 32 hpi in WT-infected dLNs (Figure 7A). Additionally, by 48 hpi, NK cells from WT-infected dLNs had significantly greater expression of KLRG1 (Figure 7B), CD137 (Figure 7C), GzmB (Figure 7D), and IFN (Figure 7E), suggesting that the initial impact of C15 on NK function occurs prior to 48 hpi. Therefore, we chose 24 and 48 hpi as timepoints for scRNA-seq, specifically probing transcriptional changes within T and NK cell (CD19-negative) populations (Figure S7D).

We recovered eight Leiden clusters, named according to key marker genes (Figures 7F and S7E). Across timepoints, IFN-responsive T cells and NK cells increased in proportion for both infection conditions (Figures S7F–G). Notably, a cluster of T cells (annotated “Activated T cells”) defined by TCR-associated genes (*Nfkb1*, *Nr4a3*, *Egr3*, *Relb*, *Dnmt3a*, *Myc*)^32,82–85^ had greater representation in C15-infected dLNs at 48 hpi (Figure S7G). Cluster-specific expression analysis did not identify DEGs 24 hpi infection across T cell clusters, but by 48 hpi, several genes downstream of TCR engagement (*Tox, Cd44, Tnf, Egr1*)^42,86,87^ and those associated with cytoskeleton remodeling (*Prag1, Tmsb10*)^88,89^ were upregulated during C15 infection (Figure 7G). In contrast, T cell clusters from WT-infected mice mainly upregulated genes associated with IFN signaling (*Mx1*, *Ifit3b*, *Socs3*). Together, these data are consistent with earlier TCR engagement in C15-infected dLNs.

When investigating NK cells at 24 hpi, only a single DEG, *Gab2* (a signaling scaffold)^90^, was upregulated in C15 dLNs (Figure 7H), and there were no DEGs at 48 hpi (Figure S7H), in contrast with phenotypic differences seen by flow cytometry (Figure 7B–E). Therefore, we performed sub-clustering on NK cells to investigate key NK cell subsets. This yielded two primary clusters (NK-1 and NK-2) (Figure 7I), with NK-1 enriched for conventional NK cell marker genes (*Ncr1*, *Klrk1*, *Gzma*, *Itga2*) and NK-2 enriched for genes associated with transcriptional regulation as well as NKT-like markers (*Tcf7*, *Lef*, *Bcl11b*)^91–94^ (Figure 7J). At 48 hpi, DEGs were only found within NK-2 (Figure 7K). WT-derived NK-2 cells had greater expression of *Klrg1* as well as several genes related to MAPK-ERK signaling (*Ksr1*, *Map3k5*, *Ndrg3*)^95–97^, regulation of apoptosis (*Hipk2*, *Wwox*)^98,99^ and vesicle transport (*Vps37b*)^100^. In contrast, C15-derived NK-2 cells had increased expression of three genes associated with regulation of calcium signaling (*Ahnak*)^101^, glycerol-3-phosphate shuttling (*Gpd2*), and actin bundling (*Lcp1*)^102^. Together, these data identify transcriptional differences in NK cells from WT- and C15-infected dLNs that may point to the key function of C15 during early infection, including a potential signaling scaffold (*Gab2*) downstream of C15-associated inhibition of NK cell function.

### C15 antagonizes NK or T cells depending on host immunocompetence

**Figure 8.**
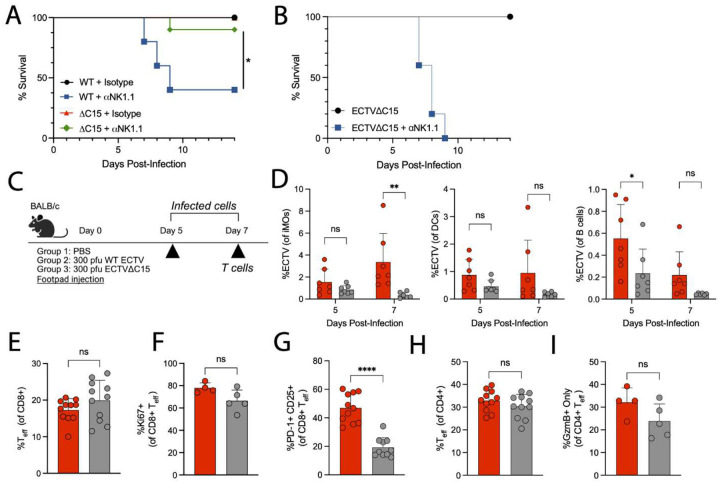
C15 antagonizes NK or T cells depending on host immunocompetence. (A) Survival curve of C57Bl/6 mice infected with WT or C15 pre-treated with isotype or NK1.1-depleting antibody. (B) Survival curve of BALB/c mice infected C15 pre-treated with isotype or NK1.1-depleting antibody. (C) Schematic for D. (D) %A33R+ (mean SD) of splenic iMOs, DCs, and B cells 7 dpi with WT or C15. (E-I) %T_eff_ of total splenic CD8+ T cells (E), %Ki67+ of CD8+ T_eff_ (F), %PD-1+CD25+ of CD8+ T_eff_ (G), %T_eff_ of total splenic CD4+ T cells (H), and %GzmB+ only of CD4+ T_eff_ (I) (mean SD) at 7 dpi with WT or C15. (A) Data derived from n=10 mice per group. (B) Data derived from n=5 mice per group. (D-I) Each dot represents an individual mouse. (A) Mantel-Cox test. (D) Two-way ANOVA with Fisher’s LSD test. (E-I) Unpaired t-test.

The genetic factors underlying the resistance of B6 mice to lethal ECTV infection, compared to other mouse strains, have been directly mapped to the natural killer complex (NKC)^103,104^. The essential role of C15-mediated NK cell inhibition to permit enhanced viral dissemination suggests that C15-NK cell engagement is the predominant NK-dependent host-pathogen interaction governing ECTV pathogenesis in B6 mice. To directly test this notion, we evaluated mortality of B6 mice infected with WT or C15 that had been pre-treated with anti-NK1.1 depleting antibody, a treatment that induces significant mortality during WT infection^27^. By 9 dpi, 60% of WT-infected mice succumbed to infection (Figure 8A). However, NK-depleted mice had significantly less mortality following C15 infection, with only a single mouse succumbing to infection 9 dpi (Figure 8A). Thus, NK cell-mediated control of infection in B6 mice is significantly less critical for protection in the absence of C15.

Other mouse strains, such as BALB/c mice, are not protected from lethal ECTV infection, with differences in susceptibility thought to be strongly driven by deficiencies in NK cell responses to infection^105,106^. This suggests that C15 engagement of NK cells may have less importance in driving pathogenesis, as NK cell responses are naturally impaired. Furthermore, previous work in BALB/c mice demonstrated that at 4 dpi, which is past the critical timepoints of early NK cell engagement, viral loads of WT and C15 are equivalent in the dLN, spleen, and liver^16^. To evaluate the importance of C15-NK cell interactions to ECTV pathogenesis, we similarly treated BALB/c mice with anti-NK1.1 antibody prior to infection with C15. Here, in contrast to B6 mice, NK cell depletion prior to infection of BALB/c mice with C15 resulted in 100% mortality (Figure 8B), indicating that, in this case, NK cell engagement by C15 is not the primary form of antagonism to host immunity. We therefore hypothesized that C15 may have a more pronounced inhibitory impact on the development of T cell responses in BALB/c mice compared to B6 mice. To test this, we infected BALB/c mice with either WT or C15 and quantified infected cells within the spleen at 5 and 7 dpi as well as T cell responses at 7 dpi (Figure 8C). In line with previous reports demonstrating lower viral titers by 6 and 8 dpi during C15 infection^16^, inflammatory monocytes (iMOs), DCs, and B cells were either trending or significantly less infected 7 dpi in C15-infected mice (Figures 8D and S6D). However, the magnitude of CD8+ T_eff_ responses were comparable between infection conditions at 7 dpi (Figures 8E), with some C15-infected mice having levels of CD8+ T_eff_ greater than WT-infected mice. While proliferation among CD8+ T_eff_ was similar as measured via %Ki67+ (Figure 8F), C15-derived CD8+ T_eff_ had lower CD25 expression (Figure 8G). Within the CD4+ T cell compartment, T_eff_ as well as CD4-CTL responses were similar between infection conditions at 7 dpi (Figures 8H–I). Collectively, these data indicate that in hosts where C15-mediated antagonism of NK cells is not the primary mode of immunomodulation, C15 does, in fact, meaningfully inhibit T cell responses, as evidenced by the equal (trending towards greater) magnitude of T_eff_ responses during C15 infection but lower viral load at 7 dpi. Furthermore, these data demonstrate the ability of C15 to exhibit “conditional antagonism”, where different inhibitory activities are exhibited, depending upon the immunological capabilities of the host.

## Discussion:

The continued global persistence of OPXVs, post-eradication of variola, highlights an incomplete understanding of their biology and a critical need for innovative antiviral strategies^107–112^. The immunomodulatory proteins encoded by OPXVs ultimately determine pathogenesis^113^, but there is much to be learned about how these proteins contribute to pathogenesis. To date, there have been limited studies on both the function and *in vivo* significance of B22 proteins^15–17,19^, despite their conservation, extreme size notwithstanding, in all highly virulent OPXVs. Our studies on the ECTV B22 protein, C15, have demonstrated its capacity to antagonize three immune cell populations: CD4+ T cells^17^, NK cells^19^, and, as shown here, CD8+ T cells. While C15 potently inhibited CD4+ and CD8+ T cells *in vitro* and *ex vivo* (Figure 1), in B6 mice, WT (C15-expressing) ECTV infection generated significantly more robust T cell responses, as indicated by phenotypic, functional and transcriptomic analyses (Figures 2, 3 and 5). We determined this was partially explained by compensatory cDC1-mediated cross-presentation (Figure 4) but primarily driven by antigen load differences from differential restriction of viral replication by NK cells (Figure 6). Specifically, early restriction of C15 viral replication by NK cells, which is antagonized by C15, dampened T cell responses, and this deficiency could be restored through the depletion of NK cells prior to C15 infection. Single cell RNA sequencing analysis identified a candidate signaling adapter, *Gab2*, that may be downstream of C15-mediated antagonism of NK cells (Figure 7). Lastly, we found that in BALB/c mice, which have genetic deficiencies in NK cell responses to ECTV, C15 more prominently inhibited T cell responses (Figure 8).

Unlike other viral virulence factors that have been described to inhibit both T cells and NK cells^23,114^, C15 does not appear to modulate the expression of key ligands critical for effector cell engagement (e.g., MHC molecules)^23,114–116^, setting it apart. All current evidence suggests that C15 inhibits cell-cell contacts^17,19^, though this has yet to be explicitly demonstrated. While parallels can be drawn between the function of synaptic events for CD8+ T cells and NK cells^117–119^, CD4+ T cells are more distinct, both kinetically^120–123^ and in post-synaptic function^124^. This raises the question of whether C15 inhibitory functions map to a single domain or are spatially separated. B22 proteins are unusually large (e.g., C15 is 1924 amino acids), and previous studies have identified two cleavage-dependent fragments^15,18^, leading to speculation that one fragment mediates each respective inhibitory activity (i.e., NK cells and T cells). Our lab is actively investigating the molecular mechanisms and structure-function relationships of C15.

Many viral virulence factors have been described to have multiple functions, and in many cases, multi-functionality is a necessity due to limited genome size. However, OPXVs are some of the largest mammalian viruses by genome size (~170–230 kb). The integration of multiple immune-targeting functions coupled with the maintenance of a very large ORF raises key questions about the evolutionary benefit of B22 proteins. Here, our findings in B6 and BALB/c mice offer a potential explanation. In B6 mice, the NK cell-targeting function of C15 is the critical host-pathogen interaction, which permits enhanced viral replication and dissemination. However, the consequence to this replication is more robust antiviral T cell responses. In BALB/c mice, where NK cells have intrinsic deficits in control of viral replication, C15 more prominently antagonizes the early development of T cell responses, which likely underlies its enhanced replication at later timepoints during infection^16^. These points suggest that the conditional antagonism of NK cells or T cells allows ECTV to robustly replicate in genetically diverse hosts, using differences in host immunocompetence to enhance viral fitness. Furthermore, our data imply that enhanced T cell responses during WT infection in B6 mice is a “trade-off” for the virus, where early viral replication is more critical for spread to uninfected hosts rather than having enhanced fitness during the adaptive immune responses. In BALB/c mice, where early viral replication is not heavily restricted by NK cells, antagonism of T cells is likely more critical for viral fitness. Thus, the evolutionary benefit of B22 proteins may stem from their adaptability against host immune defenses, though this topic requires further exploration.

Our previous work highlighting the relationship between C15 and NK cells was inferential, using whole lymph node imaging to quantify the proximity of infected cells and NK cells^19^. Here, using scRNA-seq, we were able to identify a signaling scaffold, Gab2, that may be associated with C15 antagonism of NK cells. Gab2 (Grb2-associated binder 2) or its binding partner, Grb2, are involved in the regulation of lymphocyte PI3K signaling downstream of various receptors^125^, including Fc receptors^126^, activating receptors^127^, and cytokine receptors^90^. Gab2 expression has been demonstrated in both mouse and human NK cells^126,128^, but its role in NK cell signal transduction is largely unknown. Prior work suggested that Gab2 is not required for NK cell function in mice^126^, but additional studies in human NK92MI cells implicate Gab2 as a primary regulator of NK cell PI3K signaling^128^. Thus, further studies on the role of Gab2 and PI3K signaling in NK cell activation during ECTV infection and its relationship with C15 antagonism are required. At 48 hpi, C15-derived NK-2 cells had greater expression of three genes of interest: *Ahnak*, *Gpd2*, and *Lcp1*. Previous work in T cells has identified a critical role for AHNAK in regulating calcium signaling, specifically maintaining the membrane expression of calcium channels and permitting NFAT translocation into the nucleus^101^. Given that calcium flux is required for functional NK cell cytotoxicity^129^, C15-derived NK-2 cells may have an enhanced capacity for cytolysis. Further supporting this notion is the greater expression of *Lcp1*, which encodes for the actin cross-linker L-plastin (LPL)^102,130^. LPL plays a significant role in the regulation of T cell activation through the stabilization of immunological synapses^131^ as well as transport of activating receptors^132^. Together, these upregulated genes suggest that C15-derived NK-2 cells may be functionally enhanced at this timepoint relative to WT-derived cells, in line with an inhibition of NK cells by C15.

Overall, our findings underscore the complexity of B22 proteins and their diverse engagement of the host immune response. The significant homology between C15 and the mpox B22 protein^17^, as well as other human-tropic OPXVs, necessitates investigation into the conservation of function across homologs, which could reinforce the establishment of B22 proteins as next-generation OPXV antiviral targets. Moreover, the differences in host response to infection in B6 and BALB/c mice suggest that in a genetically diverse human population, the presence or absence of potential B22 protein targets in individuals (e.g., NK receptors) may significantly impact the outcome to infection, defining whether B22 proteins primarily function to inhibit innate or adaptive immune responses.

## Methods:

### Mice

Female and male C57Bl/6J (strain #000664) and BALB/cJ (strain #000651) (8–10 weeks old) were purchased from The Jackson Laboratory. *Batf3*^*−/−*^ mice (strain #013755) were initially purchased form The Jackson Laboratory and subsequently bred in-house. In all experiments, mice were age and sex-matched across infection groups, and mice ranged between 8–12 weeks old in all infection experiments. Mice were maintained in a specific pathogen-free facility at the Children’s Hospital of Philadelphia (CHOP). All experiments and procedures were approved by the Institutional Animal Care and Use Committees at CHOP.

### ECTV and CPXV Infections

ECTV-eGFP (Strain Moscow) was a kind gift from Dr. Luis Sigal^27^. ECTV C15 was previously constructed using standard homologous recombination^17^. CPXV strain Brighton Red cell lysate was ordered from BEI Resources (NR-88) and approved under USDA Veterinary Permit #611-24-185-96159. All viruses were propagated and titered as previously described^16–18^. Briefly, ECTV viruses were seeded on 143B (TK^−^) osteosarcoma cells at a multiplicity of infection (MOI) of 3 for 72 hours. Cells were then harvested and subjected to 3x freeze-thaw-vortex cycles prior to re-seeding. After scaling up (8 × T175 flask), virus was purified by ultracentrifugation at 20,000 rpm × 1 hour on a 36% sucrose cushion. Viral stocks were resuspended in 10 mM Tris pH 9.0. CPXV was purified similarly, except viral stocks were grown using BS-C-1 cells.

Purified virus was titered by plaque assay on BS-C-1 cells under an overlay of 1% methylcellulose/complete DMEM media. For mouse infection with ECTV, C57Bl/6J and *Batf3*^*−/−*^ mice were infected with 3 × 10^3^ plaque-forming units (PFU) by injection of 15 l volume in PBS into the hind footpad. Alternatively, BALB/cJ mice were infected with 3 × 10^2^ PFU into the hind footpad. For mouse infection with CPXV, C57Bl/6J mice were infected intraperitoneally (i.p.) with 2 × 10^5^ PFU. All infections were performed in the morning between 8–10 AM. Cellular analyses during infection were performed in the spleen, liver or popliteal lymph node, as indicated.

### Cell lines

All fibroblast (e.g., B6-C15, B6-GCC, UbC-C15) cell lines were derived from in-house C57Bl/6 (B6) skin fibroblasts. B6 parental fibroblasts (MHCII-) were used to generate inducible cell lines. B6 fibroblasts expressing human CIITA and BALB/c-derived I-E^d^ were used to generate the UbC-C15 cell line. These cell lines and derivative cell lines (see below) were maintained in Dulbecco’s modified eagle medium (DMEM) containing 5% (D5) or 10% (D10) fetal bovine serum, 2 mM L-glutamine, and 1x penicillin and streptomycin. 143B and BS-C-1 cell lines were cultured in D5 and D10, respectively. Where applicable, cell lines were also cultured in the presence of 2 g/ml blasticidin to maintain purity of inducible cell lines. The generation and characterization of T cell hybridomas (NP_366–374_, OVA_257–264_, M-SL9, and NA_437–451_) was previously described^133,134^. Briefly, antigen-specific T cells were fused with the partner cell line, BWZ.36/CD8, which contained an NFAT-inducible *lacZ* cassette to readout activation. T cell hybridomas were maintained in Roswell Park Memorial Institute (RPMI) medium containing 10% FBS, 2 mM L-glutamine, 1x penicillin and streptomycin and 50 M 2-mercaptoethanol (R10). Similarly, DC2.4 cells were grown and maintained in R10 medium. All cells were grown at 37 °C in a 5% CO_2_ incubator.

### Lentivirus generation and transduction

The C15-HAtag encoding lentiviral vector was generated by performing fragmented in-fusion cloning to insert the C15-HAtag cassette in between the XbaI and SalI restriction sites of the pUltra lentiviral vector (Addgene, plasmid #24129), downstream of an eGFP reporter. The CIITA-encoding lentiviral vector was generated by removing the eGFP cassette from the pUltra vector by restriction digest and inserting a gene block for human CIITA (Uniprot P33076) with an N-terminal FLAG (DYKDDDDK) tag between the AgeI and SalI restriction sites. For lentivirus production, 6 × 10^6^ 293T cells were plated in a 10-cm dish in D5 media overnight. The following day, 22 g total of endotoxin-free plasmid preparations of the glycoprotein (VSV-G, Addgene plasmid #8454), packaging components (psPAX2, Addgene plasmid #12260), and transfer vector (pUltra-derived vector) were transfected with Lipofectamine 2000 (Invitrogen) at a 0.2:1:1 ratio. To generate lipid complexes, 50 ul of Lipofectamine 2000 was added to the plasmid mixture and complexed for 15 minutes. Five ml of pre-warmed antibiotic-free D5 media was add to cells prior to complexes. After complex addition, cells were cultured for 16 hours. The next day, media was replaced with 10 ml of complete D5 media and left for 24 hours. Lentivirus was collected at 24 and 48 hours, pooled, filtered through a 0.22- m filter, precipitated using Lenti-X Concentrator (Takara Bio) according to manufacturer protocols, and resuspended in 50 l aliquots.

To transduce cells, 1 × 10^5^ cells were resuspended in 3 ml of D5 media containing 8 g/ml polybrene (Sigma-Aldrich). One 50 l aliquot of lentivirus was then added to the cell suspension, mixed by gentle pipetting, and left to complex for 5 minutes at room temperature (RT). After 5 minutes, cells were plated in individual wells of a 6-well plate and cultured overnight. Transductants were expanded up to a T75 flask prior to cell sorting for either eGFP+ (UbC-C15) or MHCII+ (CIITA) cells. A total of three sorts were performed to recover a pure, but polyclonal, population.

### Generation of stable cell lines and induction

To generate inducible expression vectors, codon-optimized C15^17^ and guanylyl cyclase C (GC-C, kind gift from Dr. Scott Waldman) were subcloned into the pSBtet-Bla vector (Addgene, plasmid #60510) between the SfiI restriction sites. For stable cell line generation, 2 × 10^6^ B6 parental fibroblasts were plated into a 10-cm dish with D5 media and cultured overnight. The next day, 1 g of inducible, gene-of-interest vectors (C15 or GC-C) were co-transfected alongside 2 g of the pCMV(CAT)T7-SB100 vector (Addgene plasmid #34879) using 9 l of X-tremeGENE HP DNA transfection reagent (Roche). Complexes were formed at room temperature for 30 minutes, replacing culture media before addition to cells. Transfections were left in the incubator for 48 hours. After incubation, cells were trypsinized, spun down, and resuspended in 10 ml of D10-Bla (D10 + 2 g/ml blasticidin) media. For each cell line, either 1 ml, 400 l, 200 l, 100 l, 50 l, or 20 l of cell suspension was plated in a final volume of 2 ml D10-Bal in a 6-well plate. Cells were left in the incubator for 1–2 weeks, replacing every 2–3 days and monitoring the development of cell “islands”. Individual cell islands were marked using a light microscope and subsequently scraped using a p200 tip into 1 ml of D10-Bla media in individual wells of a 24-well plate. Colonies were expanded from a 24-well plate into a 6-well plate and subsequently screened by flow cytometry for protein expression.

To screen clones for protein expression, clones were trypsinized and duplicated into another 6-well plate. The duplicate plate media was supplemented with 1 g/ml doxycycline hyclate (Sigma-Aldrich), and cells were induced overnight. The following day, cells were recovered from each well using a calcium-magnesium free medium and scraping, followed by gentle resuspension. Clones were then stained, using an antibody specific for the HAtag expression, either by surface staining (C15) or intracellular staining (GC-C) and analyzed using a flow cytometer. The top clone(s) from the original plate were expanded and frozen down for further use.

To induce cells for T cell hybridoma assays (see below), cell lines were trypsinized, spun down, and resuspended in complete D5 media (no blasticidin). Between 2–3 × 10^6^ cells of each cell line were transferred into two duplicate 15-ml conical vials and topped up with D5 media to ~14.5 ml. To both tubes, 150 l of 1M HEPES pH 7.4 (Gibco) was added, but to only one tube, 150 ul of 100 g/ml doxycycline (diluted 1:100 from a 10 mg/ml stock) was added for the induced condition. Conicals were then parafilmed and rotated at 37 °C end-over-end for 16–20 hours.

### Viral genome sequencing

To prepare DNA from ECTV viruses for sequencing, genomic DNA was isolated from 0.5 ml of sucrose-cushion purified viral stock using the DNeasy Blood and Tissue Kits (Qiagen) according to manufacturer protocols. Two g of genomic DNA was sent to Genewiz for library preparation and short read whole genome sequencing, collecting a total of 1 GB sequencing data for each viral sample. Reads were mapped to the ECTV Moscow (GenBank AF012825.2) genome. Genomic variants were visualized using the Integrative Genomics Viewer (IGV Version 2.19.1)^135^. ECTV-eGFP and ECTV C15 were determined to be >99.997% identical to the reference genome.

### Whole virion ELISA and anti-A33R antibody validation

To quantify the development of anti-ECTV antibodies, high-capacity ELISA plates were coated with 2 × 10^5^ PFU ECTV Moscow in 100 l PBS overnight at 4 °C. The following day, the plates were blocked (3% goat serum, 0.5 % dehydrated milk powder, 0.1% Tween-20 in PBS) for 1.5 hours at RT with 150 rpm shaking. Plates were washed with PBS-T (PBS + 0.05% Tween-20) and 100 l of serum serial dilutions were added for 2 hours at RT with 150 rpm shaking. Serial dilutions of the human anti-A35R antibody were also added to test cross-reactivity with ECTV. After removing and washing serum/antibody dilutions, plates were stained with 100 l goat anti-mouse IgG (H+L) HRP secondary antibody at 1:5000 or goat anti-human IgG (H+L) HRP secondary antibody at 1:2500 in blocking buffer for 1 hour at RT with 150 rpm shaking. Afterwards, plates were washed and 100 l of TMB SureBlue substrate (1:1 mixture of solutions A and B, SeraCare) were added to each well without shaking for 10 minutes at RT in the dark. After 10 minutes, 50 l of 2M hydrochloric acid (HCl) was added to each well, and plates were gently tapped to distribute HCl. Immediately after neutralization, plate absorbance was measured at 450 nm.

To validate cross-reactivity of anti-A35R monoclonal with ECTV A33R, B6 parental fibroblasts were infected *in vitro* with ECTV-eGFP. Briefly, two aliquots of 5 × 10^5^ cells were spun down in 1.7-ml microfuge tubes and resuspended in 100 ul PBS + 1% FBS. To one tube, 5 × 10^5^ PFU of ECTV-eGFP were added, and the tubes were left to incubate at 37 °C for 1 hour, gently resuspending with a p200 every 20 minutes. After the incubation, tubes were topped up with D5 media and spun down. Cells were resuspended in 1 ml of D5 media and subsequently plated in a 6-well plate in a final volume of 2 ml of D5 media overnight. The next day, cells were trypsinized, spun down, and transferred to a 96-well U-bottom plate for flow cytometry staining.

### Synthetic peptides

The following synthetic peptides were used: OVA_257–264_ (SIINFEKL), M-SL9 (SLQGRTLIL)^136^, NP_366–374_ (ASNENMETM), NA_437–451_ (TVDWSWPDGAELPFT)^134^, B8R_20–27_ (TSYKFESV), and EVM153_46–60_ (VKNKYMWCYSQVNKR)^137^. All peptides were obtained lyophilized from Genscript at >85% purify. Stock concentrations were made to 10 mg/ml in DMSO.

### T cell hybridoma assay

The day before a T cell hybridoma assay, cells were induced as described above. In some instances, 1 g of mRNA-LNP/1 × 10^6^ cells was added to the 15-ml conical prior to end-over-end rotation overnight. The following day, fibroblasts (APCs) were spun down and resuspended to 2.5 × 10^5^ cells/ml in R10 media. T cell hybrids were harvested from their respective flasks and resuspended to 2.78 × 10^5^ cells/ml in R10 media. To a 96-well black flat-bottom plate, 100 ul of APCs and 90 ul of T cell hybrids were plated into respective wells. When adding peptide, 10 ul of 200 g/ml synthetic peptide diluted in 0.1% BSA/PBS was added to each corresponding well (10 g/ml final concentration). In negative control wells, 10 ul of 0.1% BSA/PBS was added in absence of peptide. Co-cultures were incubated at 37 °C for 16–20 hours. After incubation, co-cultures were lysed with a substrate buffer containing 1.25% Triton X-100, 22 g/ml 4-methyl-umbelliferyl- -D-galactopyranoside (Sigma-Aldrich), 38.5 M 2-mercaptoethanol and 9 mM MgCl_2_ in PBS for 3 hours at 37 °C. After incubation, fluorescence was quantified at 365/445 nm using a microplate reader. Hybridoma assays consisted of at least three technical replicates and were performed several independent times where indicated.

For hybridoma assays with phorbol 12-myristate 13-acetate (PMA) and ionomycin, APC-hybrid co-cultures in the presence of exogenous peptide were allowed to incubate for 1 hour at 37 °C prior to addition of 50 ng/ml PMA and 1 g/ml ionomycin.

### Trans and supernatant inhibition assay

*Trans* inhibition assays were performed the same as other T cell hybridoma assays with a few modifications. Briefly, bystander cells (MHCII-negative, B6-C15 or B6-GC-C) were spun down and resuspended to 1 × 10^6^ in R10 media. A serial dilution of bystander cells was made such that concentrations were 5 × 10^4^ cells/50 l, 2.5 × 10^4^ cells/50 l, and 1.25 × 10^4^ cells/50 l. To corresponding wells, 50 l of diluted bystanders or 50 l R10 medium alone were added. APCs (MHCII-positive, B6-CIITA) were resuspended to 5 × 10^5^ cells/ml, and 50 l were added to each well. Peptide was added to a final concentration of 10 g/ml, as above, and co-cultures were incubated at 37 °C for 16–20 hours. Assay readout was performed according to standard protocols.

To test inhibitory capacity of supernatant, 5 × 10^6^ B6-C15-CIITA or B6-GC-C-CIITA cells were induced overnight in a 15-ml conical vial. The next day, induced cells were spun down and supernatant isolated. B6-CIITA cells were then resuspended to 2.5 × 10^5^ cells in either R10, B6-C15-CIITA supernatant, or B6-GC-C-CIITA supernatant. A standard T cell hybridoma assay was then setup and readout the following day.

### Generation of mRNA-LNPs

OVA mRNA-LNPs were a kind gift of Dr. Mohamed-Gabriel Alameh and are described elsewhere.^138^ Preclinical-grade mRNA encoding the and chains of I-E^d^ were made using previously published methods.^139,140^ The chain was modified to encode a covalently linked epitope (“S1”, FERFEIFPK) to ensure proper folding of I-E^d^. To maximize translation of mRNA molecules, the sequence was codon-optimized to enrich for GC content. Optimized DNA sequences were inserted into an mRNA expression vector that was a kind gift of Dr. Drew Weissman. Expression vectors were first linearized prior to *in vitro* transcription using the MEGAscript T7 Transcription Kit (ThermoFisher), substituting N1-methylpsuedouridine (TriLink) in place of uridine and co-translationally capping with the CleanCap reagent (TriLink). Synthesized mRNA was purified using cellulose to remove dsRNA byproducts, and the quality evaluated by agarose gel electrophoresis.

Equal molar ratios of I-E^d^ and chains were co-encapsulated into LNPs. The lipid mixture was composed of the SM-102 (BroadPharm), 1,2-distearoyl-sn-glycero-3-phosphocholine (DSPC, Avanti), cholesterol (Avanti), and 1,2-dimyristoyl-rac-glycero-3-methoxypolyethylene glycol-2000 (DMG-PEG-2000, Avanti) at a 50:38.5:10:1.5 molar ratio. Lipids were dissolved in ethanol and buffered in 10% v/v citric acid, pH 4.0. Subsequently, 150 g of total mRNA were diluted in citric acid buffer. The total lipid mass to RNA mass ratio was 17.5:1. Lipids were then added into diluted mRNA and rapidly mixed via P1000 pipet 15–17 times. LNPs were then immediately neutralized with an equal volume of PBS and dialyzed in PBS overnight using a 10 kDa dialysis cassette (Thermo). The following day, mRNA-LNPs were concentrated using a 10 kDa centrifugal filter (Amicon) to 0.2–0.3 mg/ml.

### Synthesis of recombinant Flt3l

For recombinant Flt3l generation, a codon-optimized (Twist Biosciences) DNA sequence for the ectodomain of human Flt3l (Met1-Ala181, Uniprot P49771) fused to a C-terminal 6x-His tag was inserted into a CMV promoter-driven expression vector via in-fusion cloning. Endotoxin-free plasmid preparations were then transfected into Expi293 cells (ThermoFisher) according to manufacturer recommendations. Briefly, 20 g of pCMV-hFtl3l-6xHis and 80 l of Expifectamine 293 were diluted into 1.5 and 1.4 ml of Opti-MEM I Reduced Serum Media, respectively (Gibco). After incubation for 5 minutes at RT, the diluted Expifectamine 293 was added into the diluted DNA, gently mixing with a p1000 pipet. Complexes were allowed to form at RT for 15 minutes. During complexation, Expi293 cells were diluted to 3 × 10^6^ cells/ml in 25 ml of Expi293 Expression Medium (Gibco). After the incubation, formed complexes were added dropwise to Expi293 cells while gently shaking to distribute. Cells were placed back into the incubator at 37 °C while shaking on an orbital shaker at 125 rpm for 18–20 hours. The following day, 150 l of Enhancer 1 and 1.5 ml of Enhancer 2 were added to the transfected cells. The cells were then placed back into the incubator and grown for an additional 4 days (5 days total expression).

To purify recombinant protein, cell cultures were spun down at 2500 × g for 10 minutes at 4 °C. The supernatant was subsequently filtered through a 0.22 m filter and concentrated down to ~10 ml using a 10 kDa centrifugal filter (Amicon). To prepare EDTA-compatible Ni-IMAC resin (Pierce) for protein capture, 0.5 ml of slurry for each purification was transferred into a 5-ml tube on ice. After settling, the supernatant was removed and the beads were resuspended in 1.5 ml of equilibration buffer (50 mM monosodium phosphate, 300 mM sodium chloride, 10 mM imidazole, pH 8.0). The equilibration buffer was removed and the concentrated Expi293 supernatant was added to the washed beads. The cell supernatant was rotated end-over-end with beads for 1 hour at 4 °C. After incubation, the supernatant was run through a disposable gravity flow column (Bio-Rad) and rinsed once with 1 column volume (CV, ~10 ml) of equilibration buffer. The settled resin was then washed with 3 CV of wash buffer (50 mM monosodium phosphate, 300 mM sodium chloride, 20 mM imidazole, pH 8.0). The bound protein was eluted with 3 × 3 ml of elution buffer (50 mM monosodium phosphate, 300 mM sodium chloride, 500 mM imidazole, pH 8.0). Elutions were pooled and dialyzed overnight using a 10 kDa dialysis cassette (ThermoFisher) in 1x PBS. The following day, dialyzed protein was concentrated to ~1 mg/ml and confirmed to be >90% pure via SDS-PAGE. Aliquots were supplemented with sucrose to 5% v/v and flash frozen in liquid nitrogen, with long-term storage at −80 °C.

### Preparation of mouse tissues (spleen, LN, liver, PBMCs/serum)

To process spleens, organs were harvested and kept in PBS on ice until processing. Spleens were manually homogenized through a 70 m cell strainer using a 1-ml syringe plunger and subsequently washed with 10 ml of FACS buffer (PBS + 1% FBS + 2 mM EDTA). Homogenates were then spun down at 4 °C and resuspended in 3 ml of ACK lysis buffer, left at RT for 3 minutes, and topped up with cold PBS before spinning again. Cell pellets were resuspended in 10 ml of FACS buffer and passed through a 40 m strainer to prepare a single cell suspension. To harvest Flt3l-derived cDC1s, processing was the same except for a pre-incubation of spleen pieces with 1 mg/ml Collagenase Type IV and 20 g/ml DNase I for 30 minutes at 37 °C prior to homogenization.

To process draining lymph nodes (LN, inguinal and popliteal), organs were harvested and gently stripped of excess fat before placing in PBS on ice. LNs were then manually homogenized through a 70 M strainer, washed with 10 ml of FACS buffer, and immediately strained through a 40 m strainer to prepare a single cell suspension.

To process the liver, the gallbladder was removed, and the organ was kept on ice in 5 ml R10 media until processing. The liver was manually homogenized through a 70 m strainer and washed with 10 ml FACS buffer. The suspension was centrifuged at RT, and the pellet was resuspended in 5 ml RT 42% isotonic Percoll (Cytiva). The suspension was then centrifuged at 800 × g for 20 minutes at RT with no brake to separate hepatocytes (top layer) and lymphocytes (pellet). After spinning, the hepatocytes and remainder of supernatant was carefully removed. ACK lysis was then performed in 5 ml final volume for 5 minutes at RT. After lysis, 5 ml of FACS buffer was added to the suspension and centrifuged at 300 × g for 3 minutes. The lymphocyte pellets were resuspended in 5 ml FACS buffer to prepare the single cell suspension.

Blood collection was through the retro-orbital vein using a capillary tube. To harvest serum, blood was transferred into serum gel tubes (Sarstedt) and spun down at 10,000 × g for 5 minutes. Serum was subsequently stored at −20 °C. To collect PBMCs, blood was collected into 200 ul of PBS + 50 mM EDTA and diluted 1:1 with R10 media prior to underlaying 500 l of Histopaque-1083 (Sigma-Aldrich). Suspensions were centrifuged at 2000 rpm × 20 minutes at RT with no brake. PBMCs were collected from the interface and transferred in a FACS tube with 4 ml of R10 media. After spinning down, cells were ACK lysed and resuspended as necessary to prepare single cell suspensions.

### IFN- ELISpot assay

To generate cDC1s for ELISpot, one female B6 mouse was injected i.p. with 10 g of recombinant Flt3l diluted up to 200 l PBS daily for 8 days. On day 9, spleens were harvested and processed into single cell suspensions. To magnetically deplete non-cDC1s, 65 × 10^6^ splenocytes were resuspended to 1 × 10^8^ cells/ml in MACS buffer (PBS + 0.1% BSA + 2 mM EDTA) and 65 ul of magnetic depletion master mix was added (anti-TER-119: 25 g/ml, anti-CD64: 50 g/ml, anti-CD19: 50 g/ml, anti-CD3: 50 g/ml, anti-NK1.1: 50 g/ml, anti-CD317: 25 g/ml, anti-Ly6G: 25 g/ml, anti-CD31: 25 g/ml, anti-Ly6C: 50 g/ml, anti-CD16/CD32: 100 g/ml, anti-CD172a: 50 g/ml, anti-CD11b: 50 g/ml). Of note, anti-F4/80 was found to remove Flt3l-generated cDCs, and the inclusion of this antibody is not recommended. The cells and antibodies were incubated on ice for 20 minutes and subsequently diluted up to 4 ml with MACS buffer before centrifugation. Splenocytes were resuspended back to 1 × 10^8^ cells/ml in MACS buffer, and 65 ul of streptavidin nanobeads (Biolegend) were added. After 20 minutes on ice, bead-bound splenocytes were diluted up to 2.5 ml with MACS buffer and magnetically separated (cDC1s were untouched), repeating magnetic purification one additional time. To infect cDC1s, cells were spun down and resuspended to 5 × 10^6^ cells/ml in PBS + 1% FBS. WT ECTV or ECTV C15 was added to an MOI of 1, and cells were infected at 37 °C for 1 hour, with gentle resuspension every 20 minutes. After incubation, cells were washed with R10 media and resuspended to 1.5 × 10^5^ cells/ml. Two hours prior to T cell addition, 100 ul of cDC1s were added to an ELISpot plate (Millipore) precoated with an IFN capture antibody according to manufacturer protocols (BD Biosciences) and incubated at 37 °C.

To generated ECTV-specific T cells, 2 female B6 mice were infected with WT ECTV for 8 days prior to spleen harvest. After spleen processing, splenocytes were pooled and CD4+ and CD8+ T cells were isolated using corresponding Mojosort isolation kits (Biolegend). The magnetic negative selection process was identical to that of cDC1s above. CD8+ and CD4+ T cells were resuspended to 1.5 × 10^5^ cells/ml and 3 × 10^5^ cells/ml, respectively, in R10 prior to plating. To corresponding wells, 100 ul of diluted T cells were added. The ELISpot plates were allowed to incubate at 37 °C for 16–18 hours. ELISpot plates were developed according to manufacturer protocols and IFN spots were quantified using a CTL Immunospot S6 Universal Analyzer.

### Flow cytometry and tetramer

For flow cytometry, viable cells were first discriminated using either Live/Dead Fixable Aqua dye (ThermoFisher) at 1:500 or Zombie UV Fixable dye (Biolegend) at 1:1000 for 10 minutes at RT. Alongside all surface stains, anti-CD16/CD32 (BioXCell) was added to a final concentration of 10 g/ml to block Fc receptors. Surface stains were performed for 30 minutes at 4 °C in FACS buffer. In panels involving transcription factors (e.g., T-bet, TCF-1), cells were fixed and permeabilized using the Foxp3/Transcription Factor Staining Buffer Set (Invitrogen) for 1 hour at RT. For all other panels, cells were fixed and permeabilized using the Cytofix/Cytoperm Fixation/Permeabilization Kit (BD Biosciences) for 20 minutes at 4 °C. All intracellular staining was performed at RT for 30 minutes. For ECTV A33R detection, unconjugated anti-MPXV A35R was included in intracellular stains, and a BV711 anti-human secondary was subsequently added for an additional 30 minutes at RT. Samples were analyzed on the BD Fortessa or Symphony A5 cytometers, and data were processed using FlowJo (BD Biosciences).

B8R tetramers were included in the extracellular stain at 2 g/ml where applicable. Notably, the B8R tetramer is most compatible with the anti-CD8 clone KT15, as clone 53–6.7 was found to cause antigen-non-specific tetramer binding. I1L tetramer staining was performed separately for 1 hour at 37 °C at 2 g/ml prior to viability staining.

### Intracellular cytokine and degranulation assay

Six hours prior to restimulation, DC2.4 cells were harvested, and two aliquots of 5 × 10^6^ cells were resuspended to 50 × 10^6^ cells/ml in PBS + 1% FBS. Cells were subsequently left uninfected or infected with C15 at an MOI of 1 for 1 hour at 37 °C, with gentle resuspension every 20 minutes. After 1 hour, cells were diluted in a 15-ml conical vial up to 15 ml with R10 supplemented with 10 mM HEPES and rotated end-over-end for five hours. For cytokine and CD107a staining, 2 × 10^6^ splenocytes were added to a 96-well U-bottom plate in 100 l R10 medium and allowed to equilibrate for 30 minutes at 37 °C. During incubation, DC2.4 cells were spun down and resuspended to 2 × 10^6^ cells/ml in R10. To corresponding wells, 50 l of uninfected or infected DC2.4 cells were added. For peptide stimulation, 50 l of 40 g/ml peptide (B8R or 963) and 8 g/ml anti-CD28 (Biolegend) in R10 were added to corresponding wells (final concentrations 10 g/ml and 2 g/ml, respectively). Splenocyte suspensions were then incubated at 37 °C for 1 hour. After 1 hour, 50 l of 20 g/ml brefeldin A, 8 M monensin, and 8 g/ml anti-CD107a (BD Biosciences) were added to each well (final concentrations 4 g/ml, 2 M, and 2 g/ml, respectively), and splenocytes were left at 37 °C for 5 additional hours. After restimulation, cells were then stained for flow cytometry as described above. Naïve splenocytes were used as a negative control.

### FACS sorting and bulk sequencing preparation

For T_eff_ and B8R+ T_eff_ sorting, 25 × 10^6^ splenocytes were viability and surface stained for CD44, CD4, CD8, B8R tetramer, CD3, and CD62L. Samples were then sorted using an Aurora CS-2 Cell Sorter (Cytek). For bulk TCR sequencing, 4 × 10^3^ B8R+ CD8+ T_eff_ were sorted into FACS buffer. For T_eff_ bulk mRNA sequencing, between 4 × 10^4^ and 1.5 × 10^5^ CD4+ or CD8+ T_eff_ were sorted into FACS buffer. After sorting, cells were centrifuged at 400 × g for 5 minutes and washed in PBS.

For TCR sequencing, B8R+ CD8+ T_eff_ were directly lysed and subjected to first-strand synthesis using the SMART-Seq Mouse TCR (with UMIs) kit (Takara) according to manufacturer instructions. Initial TCR / chain amplifications were performed according to manufacturer instructions. For amplification during PCR #2 (UDI addition), 24 cycles gave the best yield across all samples. Amplified libraries were then pooled (equimolar) and concentrated using NucleoMag NGS Clean-up and Size Select beads (Takara). cDNA concentrations were quantified using a Qubit 4 Fluorometer, and size analysis was performed using an Agilent TapeStation.

For bulk T_eff_ mRNA sequencing, cells were lysed in 200 l TRI Reagent (Zymo) and vortexed at 1000 rpm for 60 seconds. RNA was isolated using a Direct-zol RNA Microprep kit (Zymo) according to manufacturer instructions, eluting in 8 l of nuclease-free water. mRNA quality was assessed using an Agilent Bioanalyzer. RNA was converted into cDNA libraries using the Zymo-Seq SwitchFree 3’ mRNA Library Kit (Zymo) according to the manufacturer protocol. Based on RNA concentrations, library amplification PCR reactions were performed with 19 cycles for 100 ng input RNA, 20 cycles for 75 ng input RNA, and 21 cycles for 50 ng input RNA. Sample groups (based on dpi and T cell type) were pooled and concentrated using SPRIselect Beads (Beckman Coulter). cDNA concentrations were quantified using a Qubit 4 Fluorometer, and size analysis was performed using an Agilent TapeStation.

For popliteal LN mRNA sequencing, LNs were prepared as described above. Cell pellets were resuspended in 200 l TRI Reagent and vortexed at 1000 rpm for 60 seconds. RNA was isolated using a Direct-zol RNA Miniprep kit (Zymo) according to manufacturer instructions, eluting in 20 l of nuclease-free water. mRNA quality was assessed using an Agilent Bioanalyzer. RNA was converted into cDNA libraries as above, using 100 ng input RNA. Samples were pooled and concentrated using SPRIselect beads. cDNA concentrations were quantified using a Qubit 4 Fluorometer, and size analysis was performed using an Agilent TapeStation.

### Bulk TCR sequencing and analysis

Pooled TCR libraries were sent to Novogene and sequenced using the NovaSeq X Plus with paired-end 150 bp sequencing. A total of 50 GB were sequenced across sub-libraries. For all sub-libraries, the average Q30 was 90%. FASTQ files were processed and analyzed using the Cogent NGS Immune Profiler Software (Takara) according to manufacturer recommendations, with >95% of all reads mapping to either the TCR or chain. A UMI cutoff of 8 was used to ensure only high quality UMI groups were included in clonality analyses. To calculate Simpson Clonality, the proportion of individual clonotypes (on the nucleotide level) was squared, the squared proportions were then summated, and the square root of this sum was calculated as the final clonality parameter. V and J gene usage was calculated as an output of Cogent NGS Immune Profiler Software.

### Bulk RNA sequencing and analysis

Pooled 3’ mRNA libraries were sent to Novogene and sequenced using the NovaSeq X Plus with paired-end 150 bp sequencing. A total of 350 GB were sequenced across sub-libraries, for a sequencing depth of ~30 × 10^6^ paired-end reads per sample. Due to low sequence diversity, 17% PhiX was spiked in to improve run quality. All sub-libraries had a Q30 > 78%, with an average of 82%.

To pre-process sequencing data, *umi_tools*^141^ was used to extract the UMI from read 1 and assign to corresponding read 2 (UMI-tagged). Afterwards, UMI-tagged reads were trimmed to remove polyT tracts and Illumina adapter sequence. A genome index was generated with *STAR* (version 2.7.10a)^142^ using the GRCm39 mouse reference genome. Trimmed, UMI-tagged reads were then aligned against the genome index to generate BAM files. BAM files were indexed, and UMIs were subsequently deduplicated using *umi_tools*. Prior to differential expression analysis, count matrices were generated using *featureCounts*^143^. Gene labels were mapped to Ensembl gene IDs, and samples were then analyzed using *DESeq2*^144^, identifying significant genes as those with an adjusted p-value < 0.05 and a log_2_FC > 0.5. Principal component plots were generated using rlog transformed counts. Where applicable, ambient RNA content was highlighted in volcano plots, manually identified by a high abundance of erythrocyte-associated genes.

To perform gene ontology (GO) term enrichment, differentially expressed genes were analyzed using *Metascape*^145^. The most significantly enriched terms (defined by a −log_10_(q-score) > 1.3), and their corresponding q-scores, were plotted in GraphPad prism. Gene set enrichment analysis (GSEA) was performed using software distributed by the Broad Institute^146^. Relevant gene sets were downloaded from the Molecular Signatures Database available through the GSEA website (https://www.gsea-msigdb.org/gs=a/msigdb/index.jsp). When applicable, genes strongly associated with ambient RNA (i.e., hemoglobin-associated genes) were excluded from GSEA analysis to prevent confounding outputs.

### *In vivo* antibody and antiviral treatment

For *in vivo* antibody treatments, 200 g of antibody were diluted up to 200 l in PBS and administered i.p. at specified timepoints. For antiviral treatment, CDV powder was diluted to 2 mg/ml in PBS with pulse vortexing, and the solution was 0.22- m filtered to ensure sterility. Antiviral concentration was confirmed by A_280_ absorbance prior to administration. CDV was administered in a final volume of 300 l i.p., and the untreated mice received 300 l of PBS only.

### Tetramer decay assay

The tetramer decay assay was adapted from a recent publication^47^. Female B6 mice were infected with either WT or C15 for 14 days, and spleens were subsequently harvested and processed into single cell suspensions. For each spleen, CD8+ T cells were isolated from 50 × 10^6^ splenocytes using the MojoSort Mouse CD8 Isolation Kit (Biolegend) according to manufacturer instructions. For each mouse, 2–3 × 10^6^ CD8+ T cells were plated in a 96-well U-bottom plate. Cells were subsequently stained for viability and surface stained for CD4, CD8, and B8R specificity using PE and APC tetramer conjugates. After surface staining, wells were washed and split into four equal aliquots across 4 plates. Cells were then resuspended in tetramer stabilizing solution (20 g/ml anti-H-2K^b^ and 5 g/ml anti-phycoerythrin (PE) in FACS buffer) and left for either 0, 15, 90 or 180 minutes at RT. After timepoint incubations, cells were fixed and left at 4 °C until running on the cytometer. In this experiment, no differences in binding kinetics were found between APC and stabilized PE tetramers.

### RNA isolation and qPCR in popliteal LN

Female B6 mice were infected with either WT or C15 for 24 or 32 hours. After harvesting, popliteal LNs were placed into 250 l of RNALater (Invitrogen) and left at 4 °C until processing. LNs were then removed from RNALater and placed into 600 l of TRI reagent in Type F homogenization tubes (Macherey-Nagel). Homogenization tubes were then vortexed at speed “4” at RT for 10 minutes. Homogenates were removed from beads and purified using the Direct-Zol RNA Miniprep kit (Zymo). RNA concentration was quantified using a Qubit 4 fluorometer and quality was evaluated using an Agilent Bioanalyzer.

RNA abundance was then quantified using the ZymoScript One-Step RT-qPCR kit (Zymo), performing reactions for *EVM003* and *GAPDH* (Table S4), according to manufacturer protocols. Technical duplicates were performed for each RNA sample. Relative RNA quantity was then calculated using the C_t_ method, first normalizing to *GAPDH* signal prior to calculating fold-change differences between samples. Naïve LNs were used to establish the C_t_ threshold associated with non-specific amplification (background).

### FACS sorting and fixation for scRNA-seq

For scRNA-seq, female B6 mice were infected with either WT or C15 for 24 or 48 hours. Sorting was performed on consecutive days to cut down time on ice during processing. Single cell suspensions of popliteal LNs were prepared as described above, except FACS buffer was spiked with RNasin Plus (Promega). Cells were viability stained and subsequently surface stained for CD19 at 4 °C for 30 minutes. After staining, live, CD19-positive and CD19-negative cells were separately sorted into LoBind tubes (USA Scientific) containing RNasin-supplemented FACS buffer. CD19-positive cells were pooled across infection conditions. Lymph nodes with minimal CD19-negative cell recovery were excluded from downstream analysis. Cell populations were subsequently fixed using the Evercode Cell Fixation kit (Parse Biosciences) according to manufacturer instructions. Fixed cells were concentrated, counted, and stored in either 50 or 10 l aliquots at −80 °C in Cell Storage Master Mix. One day prior to scRNA library preparation, a 10 l aliquot was thawed on ice and counted using disposable hemocytometers (Bulldog Bio). Cell counts were used to calculate the quantity of cells to load for library preparation, according to the sample loading table provided by Parse Biosciences.

### scRNA-seq sample processing and analysis

Libraries were prepared using the Evercode WT kit (Parse Biosciences), primarily following all manufacturer instructions. When performing cDNA amplification, 8 cycles were chosen based on low RNA content cells and 12,500 cells per sub-library. For size selection, SPRIselect beads were used. To perform size analysis on cDNA, the Agilent TapeStation was used. All cDNA libraries yielded >20 ng/ul in a final volume of 20 l. An initial 2 × 10^9^ paired-end 150 bp reads were generated through a promotional program with Parse Biosciences. An additional 3 × 10^9^ paired-end 150 bp reads were acquired through Novogene. Q30 values were greater than 90% for all sub-libraries with a spike-in of 10% PhiX. Across sub-libraries, a total of 69,803 cells were recovered with a total of 83,602 mean reads/cell. The median transcripts and genes per cell were 9,964 and 3,150, respectively, with a total sequencing saturation of 0.604.

The Trailmaker web server (Parse Biosciences) was used for data analysis. For quality control parameters, the automatic cell size distribution filter was applied. A manual max percentage of 3% was set for the mitochondrial content filter. An automatic spline-type fit was used to select cells with good correlation of gene vs transcript numbers. A manual probability threshold of 0.4 was set for the doublet filter. Data integration was performed with Harmony^147^ using automated settings set by Trailmaker. Lastly, cells were embedded into a UMAP and clustering was performed using the Leiden algorithm^148^, with a resolution of 0.15. For analysis focusing on T and NK lymphocytes, B lymphocyte clusters were excluded and re-clustering performed at a resolution of 0.13. The Trailmaker software was used to generate heatmaps and volcano plots visualizing differential gene expression analysis. Sub-clustering was similarly performed on NK cells (as defined by *Ncr1*), and a resolution of 0.1 was used to generate key clusters.

### Quantification and statistical analysis

GraphPad Prism was used to perform all statistical tests for cell-based assays and flow cytometry data. When comparing across multiple groups in *in vitro* data, a two-way ANOVA with Šidák correction for multiple comparisons was performed. To compare two groups (e.g., WT vs. C15) *in vitro*, a two-tailed unpaired t-test was performed. To statistically analyze *in vivo* parameters across multiple infection groups and time points (e.g., 5–10 dpi), a mixed-effects model was used with Šidák correction for multiple comparisons. For additional *in vivo* studies, two-way ANOVA with either Tukey or Šidák corrections for multiple comparisons were performed. Relevant statistical analyses are indicated within figure legends. In all experiments, results are expressed as mean standard deviation (SD). A p-value < 0.05 was considered significant, with significance indicated as *p < 0.05, **p < 0.01, ***p < 0.001, and **** p < 0.0001.

## Supplementary Material

1

Supplementary Files

This is a list of supplementary files associated with this preprint. Click to download.

- TableS1.xlsx
- TableS3.xlsx
- TableS2.xlsx
- TableS4.xlsx

Table S1. Upregulated differentially expressed genes in WT- and C15-derived CD8+ T_eff_ 7 and 10 dpi, related to Figures 2 and S2.

Table S2. Differentially expressed genes in WT- and C15-derived CD4+ T_eff_ 7 dpi, related to Figures 5 and S5.

Table S3. Differentially expressed genes in WT- and C15-infected dLNs 48 hpi, related to Figure S7.

Table S4. qPCR primer sequences for EVM003 and GAPDH quantification, related to Figure 7.

## Data Availability

Requests for further information and resources should be directed to and will be fulfilled by the lead contact, Laurence Eisenlohr (eisenlc@pennmedicine.upenn.edu). Reagents are available on request to the lead contact with a completed materials transfer agreement. All sequencing datasets are available on GEO as follows: bulk RNA sequencing i) CD4+ T_eff_ 7 dpi (GSE308806), ii) CD8+ T_eff_ 7 dpi (GSE308808), iii) CD8+ T_eff_ 10 dpi (GSE308809), iv) dLN 48 hpi (GSE309219), TCR sequencing (GSE 309222), and single cell RNA sequencing (GSE309220).
