# Supplementary material for: Conditional T and NK cell antagonism by a giant and highly conserved orthopoxvirus virulence factor
Source: Res Sq. 2026 Feb 4:rs.3.rs-8672980. Preprint. [Version 1] doi: 10.21203/rs.3.rs-8672980/v1 (PMC12889813; doi:10.21203/rs.3.rs-8672980/v1)
Supplement: 1 [file NIHPPRS8672980V1-supplement-1.pdf]

**Figure S1**

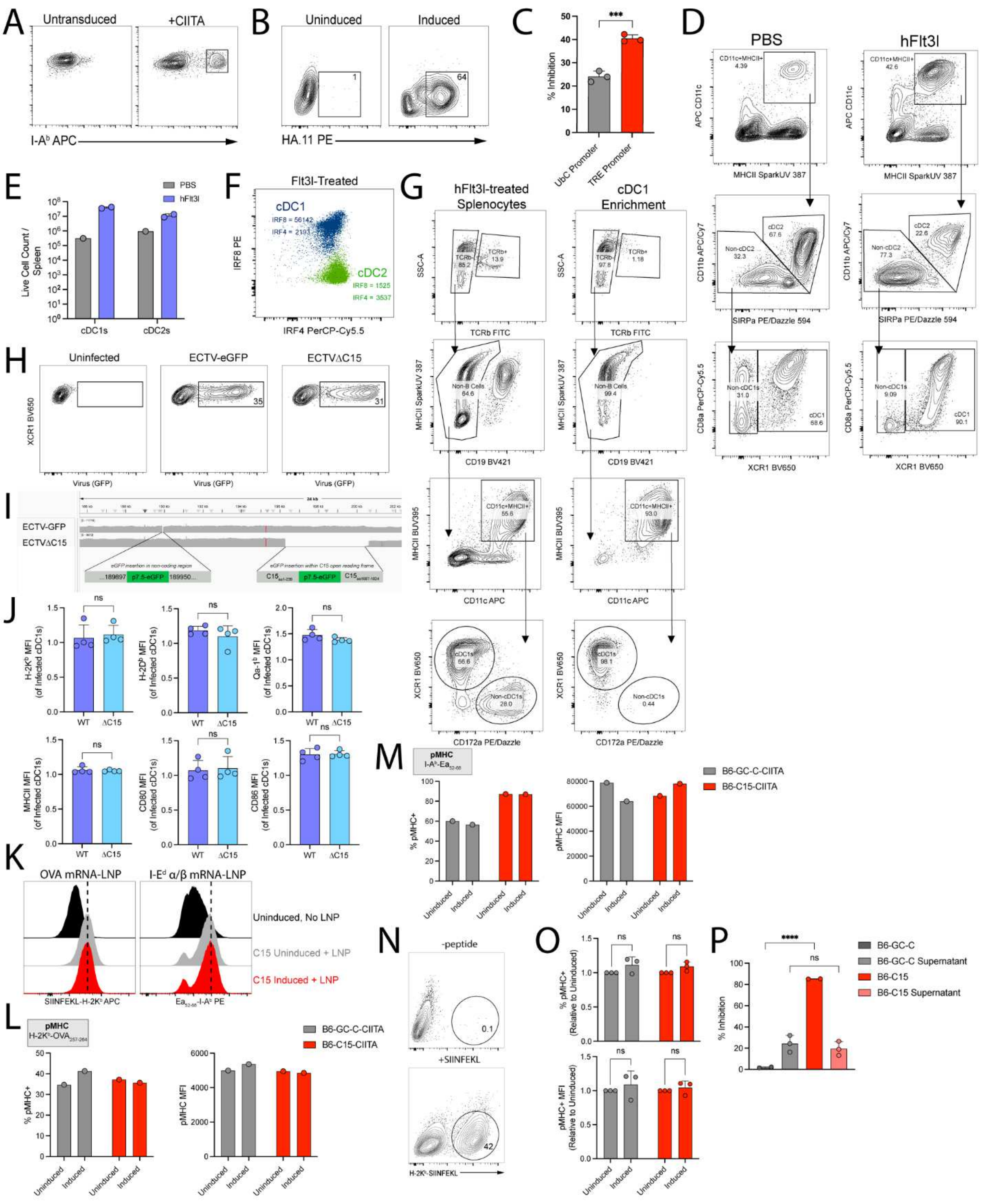


Figure S1. Validation and supporting *in vitro* data, related to Figure 1. (A) Representative flow cytometry plots showing I-A$^b$ expression of untransduced and human CIITA-transduced B6 fibroblasts. (B) Representative flow cytometry plots showing HAtag expression of uninduced and induced B6-GC-C cells. The HAtag is expressed intracellularly on the C-terminus of GC-C. (C) % Inhibition (mean  SD) of I-A$^b$-NA$_{437\text{-}451}$ hybridomas when C15 expression is driven by different promoters. (D) Flow cytometry gating strategy for cDCs. (E) Live counts of cDC1s and cDC2s per spleen for different treatment conditions. (F)

Representative flow cytometry plot of IRF4 and IRF8 expression for cDC1s and cDC2s. (G) Flow cytometry gating strategy for evaluating cDC1 purity with and without magnetic enrichment. (H) Representative flow cytometry plots of infected cDC1s across different infection conditions. (I) IGV traces of the C15 locus in ECTV-eGFP and ECTVΔC15, with key insertions highlighted. (J) MFI (mean ± SD) of various surface markers in cDC1s infected with WT or ΔC15. (K) Representative histograms of pMHC expression measured with TCR-like antibodies across different conditions. (L) %pMHC+ and pMHC MFI for LNP-derived H-2K$^{b}$-OVA$_{257\text{-}264}$ across different conditions. (M) %pMHC+ and pMHC MFI for LNP-derived I-A$^{b}$-Ea$_{52\text{-}68}$ across different conditions. (N) Representative flow cytometry plots of H-2K$^{b}$-OVA$_{257\text{-}264}$ presentation after peptide pulsing. (O) %pMHC+ and pMHC MFI (mean ± SD) of H-2K$^{b}$-OVA$_{257\text{-}264}$ after peptide pulsing across different conditions. (P) % inhibition (mean ± SD) of I-A$^{b}$-NA$_{437\text{-}451}$ hybridomas when APCs were cultured in either fresh media or conditioned media derived from high density cultures of B6-GC-C or B6-C15 cells. (C, L-M) Data derived from one independent experiment. (E) Dot represents individual mice. (J) Dot represents independent infection. (O-P) Dot represents individual experiment. (C, J) Unpaired t-test. (O) Two-way ANOVA with Sidak correction. (P) Ordinary one-way ANOVA with Tukey correction.

**Figure S2**

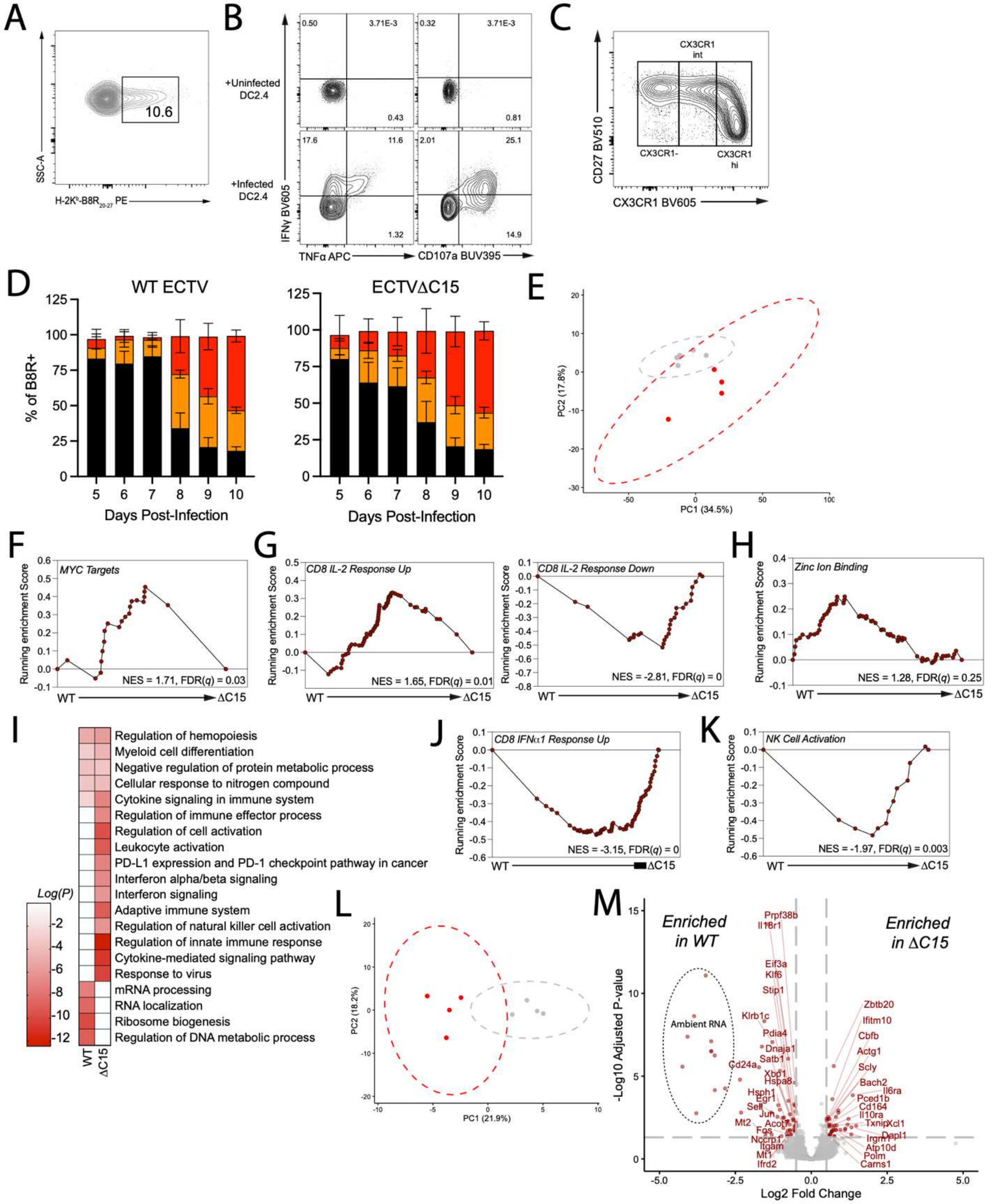


Figure S2. Additional characterization of CD8+ $T_{eff}$, related to Figure 2. (A) Representative flow cytometry plot for B8R+ CD8+ $T_{eff}$. (B) Representative flow cytometry gating for CD8+ T cells restimulated with

either uninfected or □C15-infected DC2.4 cells. (C) Representative flow cytometry plot of CX3CR1 expression in CD8+ $T_{eff}$ at 9 dpi. (D) Distribution of CX3CR1 expression (negative, intermediate, or high) on splenic B8R+ CD8+ $T_{eff}$ over time in WT- or □C15-infected mice. (E) PCA of RNA-seq of WT- or □C15-derived CD8+ $T_{eff}$ at 7 dpi. (F-H) GSEA of MYC targets (F), CD8 IL-2 response (G), and zinc ion binding (H) between WT- and □C15-derived CD8+ $T_{eff}$ at 7 dpi. (I) Enriched GO terms in CD8+ $T_{eff}$ from each infection condition. (J-K) GSEA of CD8 IFN□1 response (K), and NK cell activation (L) between WT- and □C15-derived CD8+ $T_{eff}$ at 7 dpi. (L) PCA of RNA-seq of WT- or □C15-derived CD8+ $T_{eff}$ at 10 dpi. (M) DEGs between WT- and □C15-derived CD8+ $T_{eff}$ at 10 dpi. (D) Data pooled across 1-3 independent experiments with n=4-15 for each group. (E-M) Sequencing data derived from n=4-5 per group.

# Figure S3

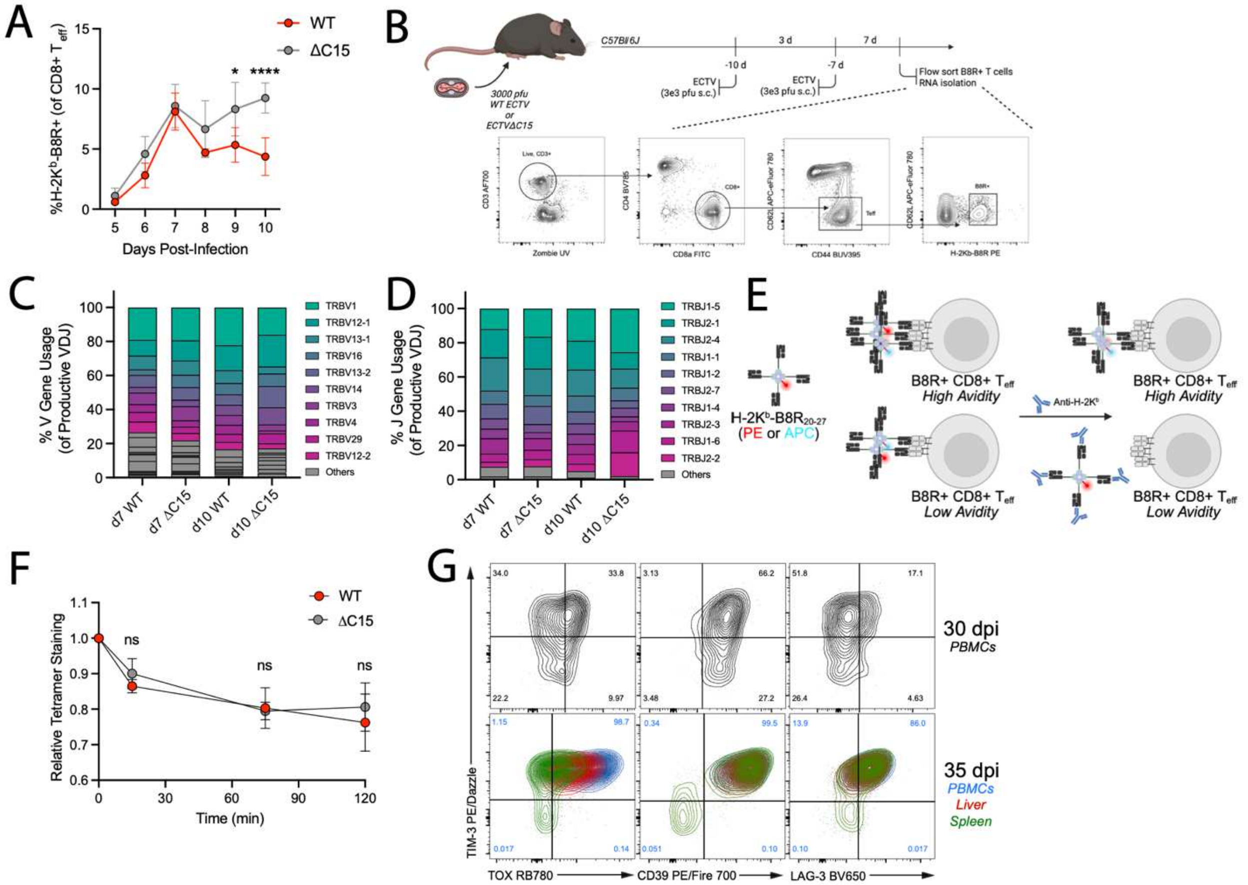


Figure S3. Supporting data for CD8+ T cell clonality and avidity, related to Figure 3. (A) Proportion of B8R+ CD8+ $T_{eff}$ (mean □ SD) over time for WT or □C15 infection. (B) Schematic for B8R+ CD8+ $T_{eff}$ FACS sorting experiment. (C) %V Gene usage of B8R+ CD8+ $T_{eff}$ across different infection conditions and timepoints. (D) %J Gene usage of B8R+ CD8+ $T_{eff}$ across different infection conditions and timepoints. (E) Schematic for adapted tetramer decay assay. (F) Relative proportion of tetramer staining (mean □ SD) for WT- and □C15-derived CD8+ $T_{eff}$ over time. (G) Representative flow cytometry plots for various exhaustion markers in B8R+ CD8+ $T_{eff}$ derived from PBMCs (30 dpi or 35 dpi) or liver and spleen (35 dpi). (A) Data pooled across 1-3 independent experiments with n=4-15 for each group. (F) Data derived from n=5 per group. (A) Mixed-effects model with Sidak correction. (F) Two-way ANOVA with Tukey correction.

## Figure S4

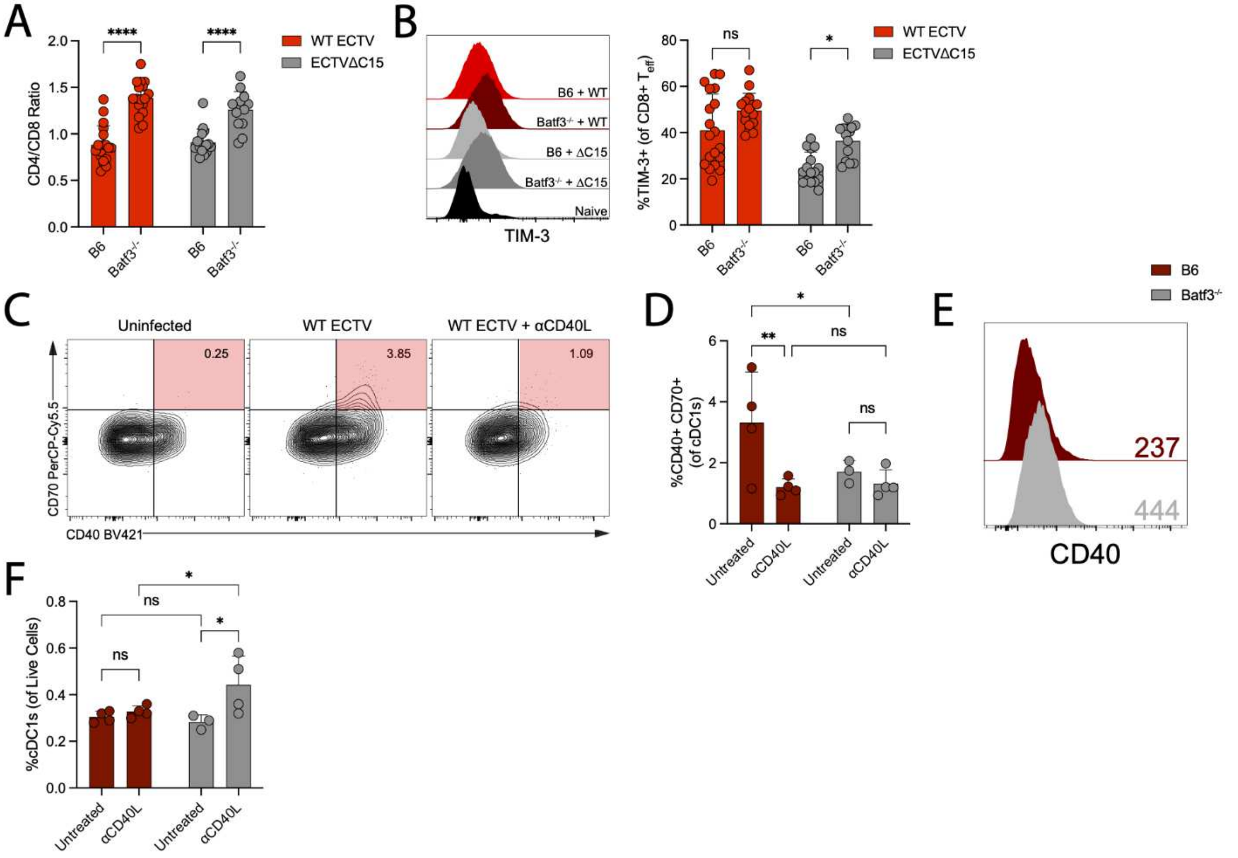


<u>Figure S4. Supporting data for ECTV infection of *Batf3-/-* mice, related to Figure 4.</u> (A) CD4/CD8 ratio (mean  SD) of CD3+ splenocytes from WT- or C15-infected B6 or *Batf3-/-* mice 7 dpi. (B) Representative histogram and quantification of TIM-3+ (mean  SD) CD8+ $T_{eff}$ derived from WT- or C15-infected B6 or *Batf3-/-* mice 7 dpi. (C) Representative flow cytometry plots of CD70 expression in cDC1s 7 dpi. (D) %CD40+ CD70+ (mean  SD) of cDC1s from WT-infected B6 or *Batf3-/-* untreated or treated with CD40L blocking antibody 7 dpi. (E) Representative histogram of CD40 expression on cDC1s derived from B6 or *Batf3-/-* mice 7 dpi. (F) %cDC1s of live cells (mean  SD) from WT-infected B6 or *Batf3-/-* mice untreated or treated with CD40L blocking antibody 7 dpi. (A-B) Data pooled across three independent experiments. (A-B, D, F) Dot represents individual mouse. (B) Data derived from n=4-15 mice per group. (A-B) Two-way ANOVA with Tukey correction. (D, F) Two-way ANOVA with Fisher’s LSD test.

**Figure S5**

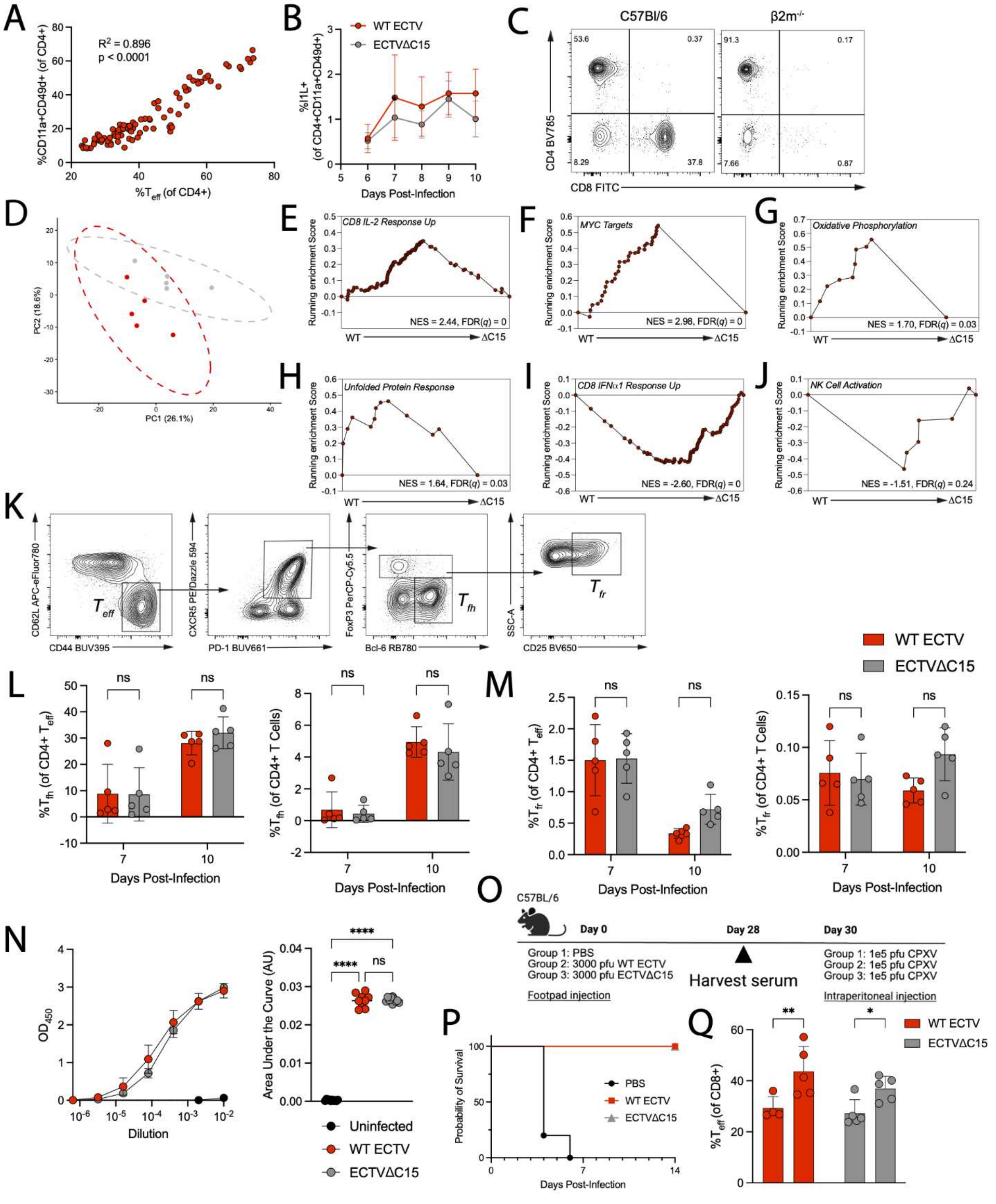


<u>Figure S5. Supporting data for CD4+ T cells responses and cross-protective immunity, related to Figure 5.</u> (A) Correlation plot of CD11a+ CD49d+ CD4+ T cells and CD4+ $T_{eff}$. (B) %I1L+ (mean □ SD) of CD11a+ CD49d+ CD4+ T cells for each infection condition. (C) Representative flow cytometry plot of CD3+ splenocytes in C57Bl/6 and □2m$^{-/-}$ mice. (D) PCA of RNA-seq of WT- or □C15-derived CD4+ $T_{eff}$ at 7 dpi. (E-J) GSEA of CD8 IL-2 response (E), MYC targets (F), oxidative phosphorylation (G), unfolded protein response (H), CD8 IFN□1 response (I), and NK cell activation (J) between WT- and □C15-derived CD4+ $T_{eff}$

at 7 dpi. (K) Flow cytometry gating strategy for $T_{fh}$ and $T_{fr}$. (L-M) %$T_{fh}$ (L) or %$T_{fr}$ (M) (mean ± SD) of total CD4+ T cells or CD4+ $T_{eff}$ in iLN at 7 and 10 dpi in different infection conditions. (N) ELISA $OD_{450}$ (mean ± SD) of diluted serum and quantified area under the curve 28 dpi for each infection condition. (O) Schematic for P-Q. (P) Survival curve of differentially immunized mice challenged with 1 x $10^5$ pfu of cowpox virus i.p. (Q) %$T_{eff}$ (mean ± SD) of CD8+ T cells across immunization and challenge conditions. (A-B) Data pooled across 1-3 independent experiments with n=4-15 for each group. (D-J) Sequencing data derived from n=5 mice per group. (N) Data derived from n=10 per group. (P) Data derived from n=5 per group. (A, L-N, Q) Dot represents individual mouse. (A) Pearson correlation. (L-M) Two-way ANOVA with Sidak correction. (N) Ordinary one-way ANOVA with Tukey correction. (Q) Two-way ANOVA with Fisher’s LSD test.

**Figure S6**

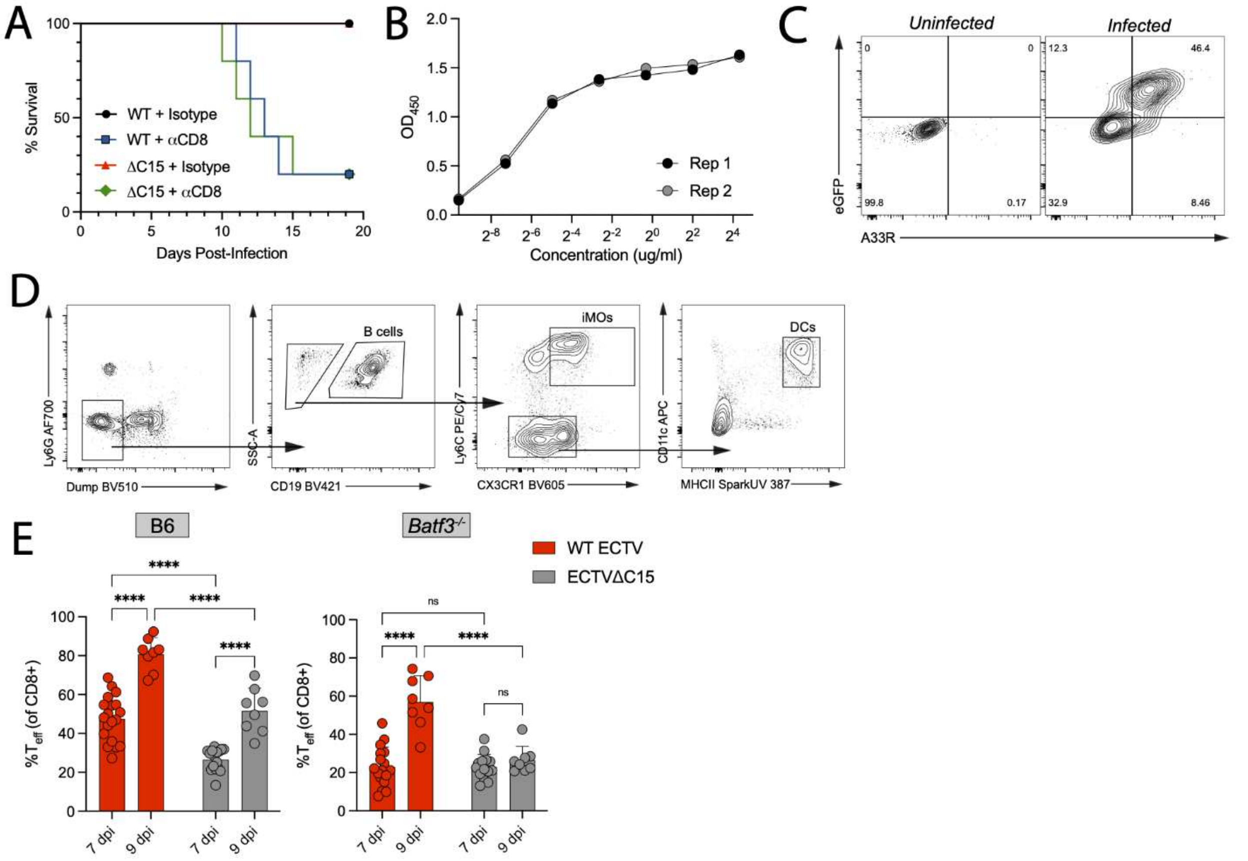


<u>Figure S6. Supporting and validation data related to Figure 6.</u> (A) Survival curve of C57Bl/6 mice infected with WT or ΔC15 pre-treated with isotype or CD8-depleting antibody. (B) ELISA $OD_{450}$ of diluted anti-A35R (A33R) antibody across two replicates. (C) Representative flow cytometry plots of eGFP and A33R co-expression for uninfected or WT-infected B6 fibroblasts. (D) Flow cytometry gating strategy for splenic B cells, iMOs, and DCs in infected mice. (E) %$T_{eff}$ (mean ± SD) of splenic CD8+ T cells from WT- or ΔC15-infected B6 or *Batf3*$^{-/-}$ mice 7 and 9 dpi. (A) Data derived from n=5 per group. (E) Data pooled across 4 independent experiments. (E) Dot represents individual mouse. (E) Two-way ANOVA with Tukey correction.

**Figure S7**

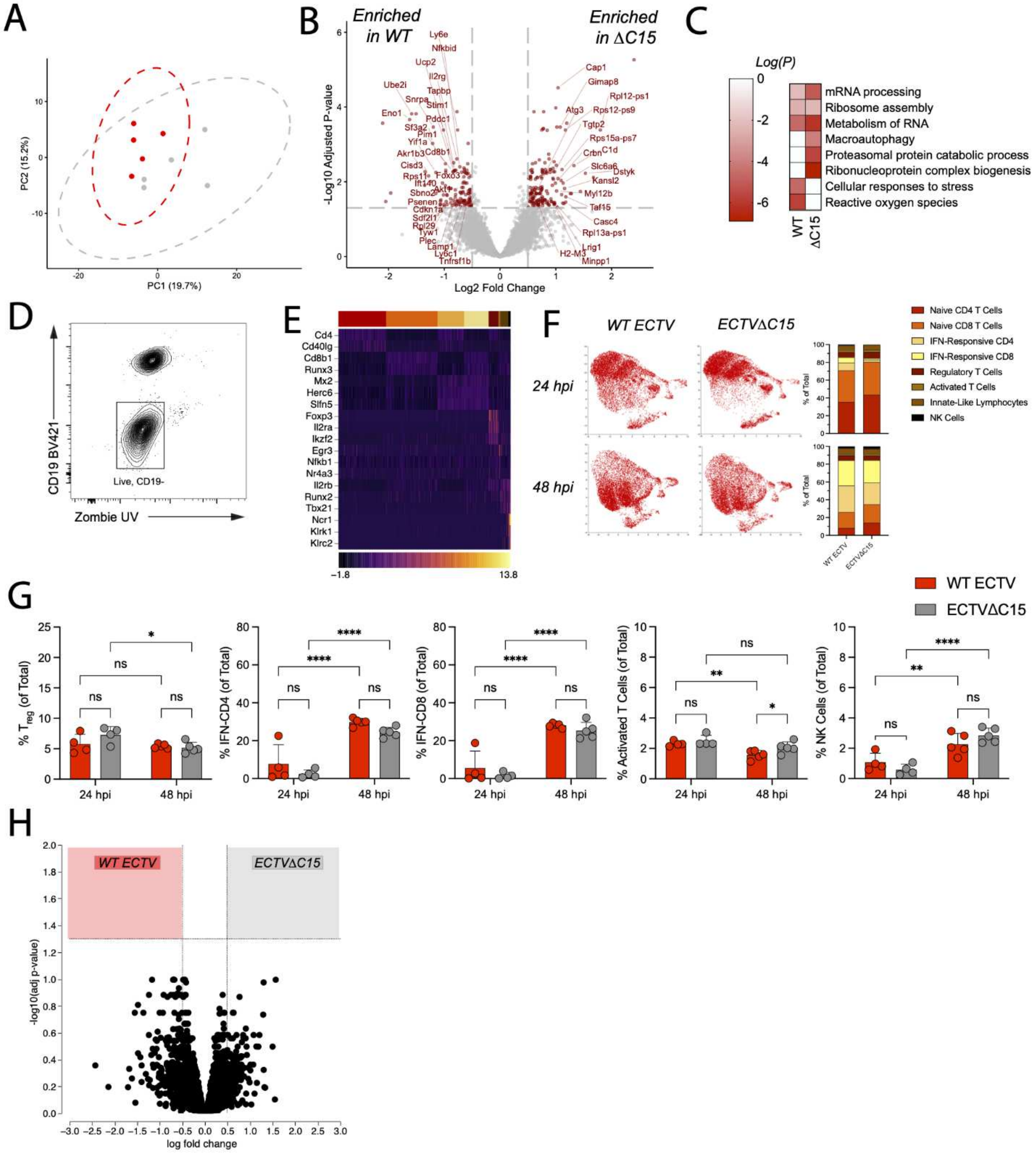

Figure S7. Supporting data for transcriptomic analysis of dLN, related to Figure 7. (A) PCA of RNA-seq of WT- or C15-infected dLNs at 48 hpi. (B) DEGs between WT- and C15-infected dLNs at 48 hpi. (C) Enriched GO terms in dLNs from each infection condition. (D) FACS gating strategy to isolate CD19- cells from dLNs. (E) Expression heatmap of key marker genes for each Leiden cluster. (F) Projection of cells

derived from 24 or 48 hpi onto the pooled UMAP and cluster proportions at each time point. (G) Leiden cluster proportions (mean ± SD) for WT- or ΔC15-infected dLNs at 24 or 48 hpi. (H) DEGs of NK cells from WT- and ΔC15-infected dLNs at 48 hpi. (A-C, E-H) Sequencing data derived from n=4-5 per group. (G) Dot represents individual mouse. (G) Two-way ANOVA with Fisher's LSD test.
